# Concurrent and discriminant validity of ActiGraph waist and wrist cut-points to measure sedentary behaviour, activity level, and posture in office work

**DOI:** 10.1186/s12889-021-10387-7

**Published:** 2021-02-12

**Authors:** Roman P. Kuster, Maria Hagströmer, Daniel Baumgartner, Wilhelmus J. A. Grooten

**Affiliations:** 1 Division of Physiotherapy, Department of Neurobiology, Care Sciences and Society, Karolinska Institutet, Stockholm, Sweden; 2 IMES Institute of Mechanical Systems, School of Engineering, ZHAW Zurich University of Applied Sciences, Winterthur, Switzerland; 3 Medical Unit Occupational Therapy and Physiotherapy, Allied Health Professionals, Karolinska University Hospital, Stockholm, Sweden; 4 Department of Occupational Therapy & Physiotherapy, Theme Women’s Health and Allied Health Professionals, Karolinska University Hospital, Stockholm, Sweden

**Keywords:** Activity-promoting chair, Agreement, Counts-per-minute, Kappa, Physical activity, ROC curve, Sit-stand desk, Workplace intervention

## Abstract

**Background:**

Sedentary Behaviour (SB) gets an increasing attention from ergonomics and public health due to its associated detrimental health effects. A large number of studies record SB with ActiGraph counts-per-minute cut-points, but we still lack valid information about what the cut-points tell us about office work. This study therefore analysed the concurrent and discriminant validity of commonly used cut-points to measure SB, activity level, and posture.

**Methods:**

Thirty office workers completed four office tasks at three workplaces (conventional chair, activity-promoting chair, and standing desk) while wearing two ActiGraphs (waist and wrist). Indirect calorimetry and prescribed posture served as reference criteria. Generalized Estimation Equations analysed workplace and task effects on the activity level and counts-per-minute, and kappa statistics and ROC curves analysed the cut-point validity.

**Results:**

The activity-promoting chair (*p* < 0.001, ES ≥ 0.66) but not the standing desk (*p* = 1.0) increased the activity level, and both these workplaces increased the waist (*p* ≤ 0.003, ES ≥ 0.63) but not the wrist counts-per-minute (*p* = 0.74) compared to the conventional chair. The concurrent and discriminant validity was higher for activity level (kappa: 0.52–0.56 and 0.38–0.45, respectively) than for SB and posture (kappa ≤0.35 and ≤ 0.19, respectively). Furthermore, the discriminant validity for activity level was higher for task effects (kappa: 0.42–0.48) than for workplace effects (0.13–0.24).

**Conclusions:**

ActiGraph counts-per-minute for waist and wrist placement were – independently of the chosen cut-point – a measure for activity level and not for SB or posture, and the cut-points performed better to detect task effects than workplace effects. Waist cut-points were most valid to measure the activity level in conventional seated office work, but they showed severe limitations for sit-stand desks. None of the placements was valid to detect the increased activity on the activity-promoting chair. Caution should therefore be paid when analysing the effect of workplace interventions on activity level with ActiGraph waist and wrist cut-points.

**Supplementary Information:**

The online version contains supplementary material available at 10.1186/s12889-021-10387-7.

## Background

Sedentary Behaviour (SB), defined as sitting or reclining with ≤1.5 Metabolic Equivalents (MET) [1], is a substantial part of the modern lifestyle, accounting for 8.5–10 h a day or 60–70% of waking time [2–4]. Up to 72 and 81% of the European Union and United States population works in the tertiary, predominantly office based sector [5]. Office workers spend around 64–82% of their working time sedentary [6–8], and accumulate half of their sedentary time during working hours, predominantly in long bouts [9, 10]. Thus, office work is of critical importance for public health, and some public health authorities included SB in their physical behaviour recommendations [11], but office workers show bad adherence [10].

The use of workplace interventions to break up prolonged SB, like standing desks and activity-promoting office chairs [12], is highly recommended [13]. These interventions typically address one of the two components of SB: 1) break up prolonged sitting with standing, or 2) break up prolonged minimal-intensity physical activity (minPA, ≤1.5 MET [14]) with light-intensity physical activity (LPA, > 1.5 MET) (operational definitions are given in Table 1).

**Table 1:** Operational definitions of behaviour, activity level, and body posture

Terminology	Definition
**Behaviour**	
Sedentary Behaviour (SB)	Sitting or reclining with ≤1.5 Metabolic Equivalents^a^
Non-Sedentary Behaviour (non-SB)	All tasks not fulfilling the SB definition
**Activity Level**	
Minimum-intensity physical activity (minPA)	≤1.5 Metabolic Equivalents, regardless of posture^b^
Light-intensity physical activity (LPA)	1.5–3.0 Metabolic Equivalents
**Body Posture**	
Sitting	Resting on the buttocks or haunches^c^
Standing	Supporting oneself on the feet in an erect position^c^

^a^as defined by the Sedentary Behavior Research Network [1]

^b^as introduced by Holtermann et al. [14]

^c^as defined by the Merriam-Webster Dictionary [15, 16]

To break up the posture component of SB, there is some evidence that sit-stand desks reduce the sitting time by around 100 min a day in short-term (≤3 months) and 60 min a day in mid-term (3 to 12 months) compared to sit-desks [17, 18], without compromising user comfort and productivity [19, 20]. If combined with other interventions like counselling, prompts or social support, the effect might remain also in long-term (> 12 months) [21, 22]. In contrast, interventions to break up the activity component of SB show inconsistent findings [18, 23], with potentially negative effects on work performance [19]. An activity-promoting chair showed a 20% activity increase compared to normal sitting [24], with unknown short-, mid- and long-term effects [18, 25].

Unfortunately, the evidence for the effects of workplace interventions is mixed, which could be related to, among other things, the use of inconsistent SB measurements [17, 18, 22, 25]. While self-reported measures are known to have a poor validity [26, 27], the objective methods to measure SB use two different principles: accelerometers worn on the thigh using the sensor orientation versus gravity to determine the posture component of SB (i.e. activPAL, PAL Technologies, Glasgow, SCO), and accelerometers worn on the waist using a proprietary counts-per-minute (cpm) measure to determine the physical activity component of SB (i.e. ActiGraph, ActiGraph Corp., Pensacola, USA) [17, 18, 22]. Posture-based devices are highly valid to measure sitting and should therefore be the first choice to analyse the effects of workplace interventions targeting posture, i.e. reducing the time spent sitting [28, 29]. On the other hand, it is unclear what the activity-based devices tell us about office work. A recent review on daily sedentary patterns included 64 studies, of which 43 used a waist-worn ActiGraph with a cut-point of 50, 100, or 150 cpm [30]. To measure SB, cut-points between 25 and 250 cpm for the vertical axis (VA) and between 100 and 200 cpm for the vector magnitude (VM) are used [31]. However, to measure minPA, the activity component of SB, VA cut-points between 50 and 200 cpm are recommended [32, 33], and to measure sitting, the posture component of SB, VA cut-points between 22 and 150 cpm are recommended [29, 34–36]. The ActiGraph is nowadays also worn at the wrist, with a recommended VM cut-point to detect sitting of 1′853 cpm [36].

Overall, it remains unclear what all these ActiGraph cut-points tell us about office work and workplace interventions, and whether they measure behaviour, activity, or posture (Table 1). The present study therefore analysed the concurrent validity of commonly used waist and wrist cut-points to detect SB, minPA, and sitting, as well as the discriminant validity of commonly used waist and wrist cut-points to separate SB from non-SB (behaviour classification), to separate minPA from LPA (activity classification), and to separate sitting from standing (posture classification).

## Methods

### Study population

A convenience sample of 30 office workers from the local community with at least 70% employment rate, all spending at least 50% of their work time at an office desk was recruited. Office workers with silicon allergy were excluded. The participants (53% women) averaged 38.8 ± 9.0 years, 71.2 ± 11.1 kg, and 1.74 ± 0.08 m. They worked on average 40.5 ± 6.6 h a week, of which 78 ± 15% seated at an office desk (self-reported).

### Experimental procedure and equipment

**Fig. 1:**
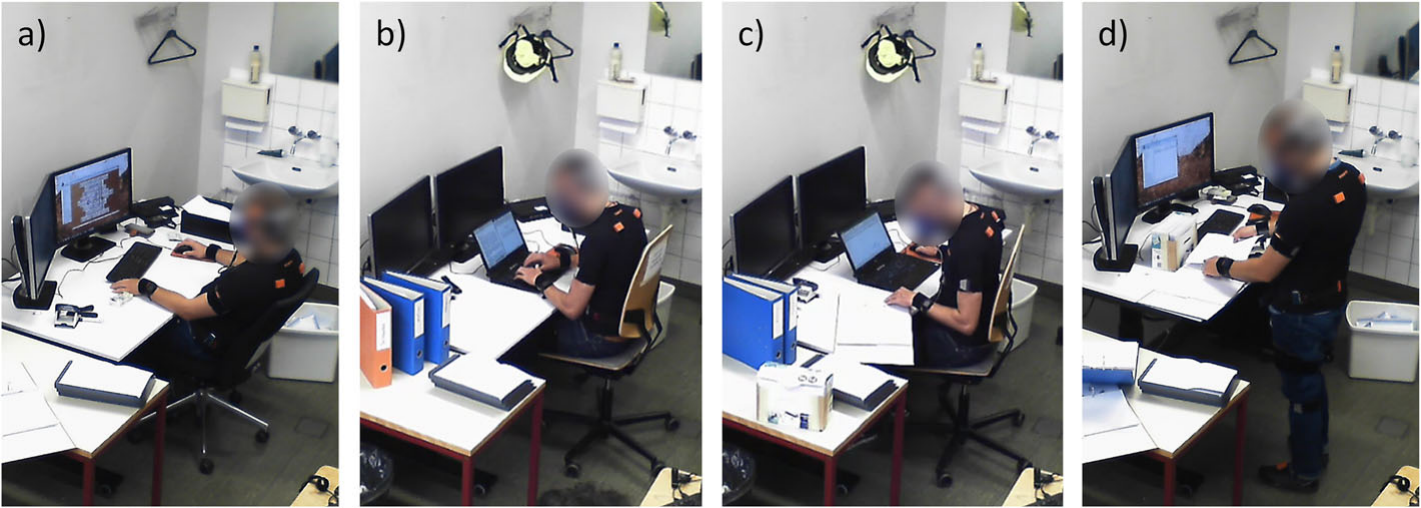
Investigated workplaces and tasks. Participant using the conventional chair (**a**), the activity-promoting chair (**b** and **c**), and the standing desk (**d**) to complete the four tasks Mouse (**a**), Keyboard (**b**), Deskwork (**c**), and Sorting (**d**). Picture **b** and **c** show a participant using the laptop

All participants visited a single office at the University in Winterthur (SUI) normally used by the researcher in charge of data recording. For the indirect calorimetry measurement, they refrained from eating and consuming sugary and caffeinated drinks for 2 h prior to the measurement, from any kind of physical activity for 12 h, and from nicotine for 2 h [37, 38]. The office was equipped with a sit-stand desk, a laptop with external mouse, a desktop computer with two screens, and two office chairs (a conventional and an activity-promoting chair). To account for different workplace designs, we pragmatically decided to let participant 15 to 24 use a laptop, while the others used a desktop computer (Fig. 1).

Each participant completed four office tasks with different activity levels [12, 39]. Two tasks were computer-assisted (Mouse and Keyboard), one task was partly computer-assisted (Deskwork), and one task was non-computer-assisted (Sorting). The Mouse task was playing mah-jong, the Keyboard task was writing a text, the Deskwork task included successive short tasks like getting a physical folder, search in it, do mental arithmetic, write notes, and switch screen views, and the Sorting task consisted of opening envelopes and stow the documents according to the instruction on it in specified storage compartments and physical folders. Each task was performed at three workplaces: a conventional office chair (Vitra, Birsfeld, SUI), an activity-promoting office chair (rotavis, Winterthur, SUI), and a standing desk (Fig. 1). The activity-promoting chair is described in detail elsewhere [40, 41]. In short, the chair has a moveable seat allowing the user to move the pelvis to the left and right while keeping a stable upper body position to not interfere with office work demands. An unpublished study showed that this chair has the potential to increase the activity level from minPA to LPA, and thus to break up SB. The activity-promoting chair was combined with an oral prompt to remind participants every minute to move the seat of the chair if they did not move it.

Participants were equipped with two ActiGraph accelerometers (wGT3X-BT at the left waist mounted with an elastic stripe, GT9X-Link at the left wrist mounted with a wristband) and the indirect calorimeter (K5, cosmed, Rome, Italy) with size-matched facemask. All sensors were initialised with the same computer to ensure synchronous recordings. The ActiGraphs recorded with 30 Hz and disabled idle sleep mode. The indirect calorimeter recorded with 0.1 Hz in the mixing chamber mode. Note that the study included additional sensors not used for the present analysis and thus reported elsewhere [42]. The participants completed the four tasks at each of the three workplaces in random order, ensuring that the same task and workplace never occurred in succession. Before each task was recorded, participants set up the workplace according to their preferences, but they could modify the setting at any time during the recording. Based on a pilot study in which we observed the onset of steady state energy expenditure after 4 min at the latest, all tasks were recorded for 5 min. After completion of the twelve office tasks, an additional resting measurement was conducted. The participants were lying for 10 min on a yoga mat with the office door closed and the sunblind lowered.

### Processing

ActiGraph data were downloaded with ActiLife Version 6.13.4 and exported to 1 s-epoch cpm files in csv-format using the normal and the low-frequency-extension filter. Following the recommendation to use the low-frequency-extension filter for lower intensity activities [43], only the low-frequency-extension filtered data are presented in the manuscript, the normal filtered data can be found in Additional file 1. K5 data were downloaded with Omnia Version 1.6.7 and exported as csv-file. The csv-files were loaded into Matlab 2019a for processing and analyses.

The ActiGraph 1 s-epoch cpm files were summarized into minute-by-minute data using the start information of each task. The time-matched K5 data was processed on a task-by-task level, and all ActiGraph minutes of a task were assigned to either minPA or LPA using the median steady state MET. Therefore, the respiratory gas exchange was converted into the metabolic rate using the Weir equation (metabolic rate = 1.44 [3.94 VO_2_ + 1.11 VCO_2_]) and put in relation to the resting metabolic rate. The resting metabolic rate was defined as the median metabolic rate during the second 5 min of the resting measurement [44, 45]. For each task, only steady state data was considered. The onset of steady state was defined by the first minute with less than 10% deviation from the median of all subsequent minutes, but earliest after one and latest after 4 min [42]. All minutes of each task were furthermore assigned into the prescribed posture (sitting for the conventional and activity-promoting chair, standing for the standing desk, verified by direct observation).

### Analyses

To analyse whether the MET values, the activity classification (minPA with ≤1.5 MET or LPA with 1.5–3.0 MET), and the ActiGraph’s cpm values differed between the workplaces and tasks, Generalized Estimation Equations followed by pairwise post-hoc comparisons with Bonferroni-adjusted *p*-values were used (performed in SPSS 27, IBM, Armonk, USA). Level of significance was set to 0.05. Following the recommendation to include effect sizes as a measure of importance of a statistical effect, significant MET and cpm effects were quantified with the effect size based on the Wilcoxon-matched-pair statistics (ES) [46]. No effect sizes were calculated for the activity classification as we are not aware of an established statistics to calculate effect sizes for binary classification data. The effects were considered small (0.1–0.3), medium (0.3–0.5), or strong (≥0.5) [47]. Note that the workplace effects were analysed to check whether there is an additional need to analyse the discriminant validity for effects caused by workplaces only.

The concurrent validity analysed the ability of the cpm cut-points to detect SB, minPA, and sitting over the entire data set, as well as for each workplace and task separately. The discriminant validity analysed the ability of the cpm cut-points to discriminate SB from non-SB (behaviour classification), to discriminate minPA from LPA (activity classification), and to discriminate sitting from standing (posture classification) on an individual level over the entire data set, as well as for effects caused by workplaces and tasks only. Both validities used kappa statistics with 95% confidence intervals [48] and ROC curves with sensitivity and specificity. The agreement with the reference criteria was considered poor (kappa < 0.00), slight (0.00–0.20), fair (0.21–0.40), moderate (0.41–0.60), substantial (0.61–0.80), or almost perfect (≥0.81) [49]. The waist-worn ActiGraph used cut-points between 22 and 250 cpm (VA) and between 100 and 200 cpm (VM) [31, 36]. The wrist-worn ActiGraph used cut-points between 1′000 and 3′000 cpm (VM). For a better understanding, the ROC curves include also lower (down to 0 cpm) and higher cut-points (up to 500 and 750 for waist VA and VM, up to 15′000 for wrist VM).

The analysis considered all recorded ActiGraph minutes of each participant. To test for dependence effects (each participant contributed for each workplace-task combination five subsequent minutes), the entire analysis was repeated for each minute individually (minute 1 to minute 5). Since kappa for the individual minutes lay mostly within the 95% confidence interval of the all minutes’ approach (Additional file 2), only the results of the all minutes’ approach are presented.

## Results

Of the 1′800 recorded minutes, 1 min had to be excluded due to non-compliance with the study protocol (standing for conventional chair). To simplify the analysis, the missing minute was replaced with the median cpm of the task. Across all workplaces and tasks, the participants reached the steady state MET within the first 2 min in 83.6% of the cases, after 3 min in 12.5% of the cases, and after 4 min in the remaining 3.9% of the cases.

### Workplace and task effects on the MET value and the activity classification

There was an overall workplace effect on the MET value and the activity classification (Table 2). The activity-promoting chair caused a significantly higher MET and lower minPA classification compared to the conventional chair and the standing desk (*p* < 0.001, ES ≥0.66). No differences between the conventional chair and the standing desk were found (*p* = 1.000).

**Table 2:** Comparison of the metabolic equivalent and the activity classification for all workplaces and tasks

**Tasks**		**Workplaces**				**Task Effects**			
		ConvChair	ActiveChair	Standing	Overall	Overall	vs. Keyboard	vs. Deskwork	vs. Sorting
Mouse	MET value	1.19 [1.11–1.24]	1.33 [1.25–1.45]	1.18 [1.09–1.28]	1.23 [1.18–1.35]		**0.002 (0.42)**	**0.000 (0.69)**	**0.000 (0.87)**
	Activity classification	96.7	73.3	100.0	90.0		0.132	**0.000**	**0.000**
Keyboard	MET value	1.34 [1.15–1.38]	1.40 [1.33–1.50]	1.28 [1.20–1.32]	1.30 [1.24–1.37]		–	**0.000 (0.40)**	**0.000 (0.87)**
	Activity classification	93.3	70.0	90.0	84.4		–	**0.000**	**0.000**
Deskwork	MET value	1.26 [1.20–1.41]	1.52 [1.34–1.60]	1.33 [1.26–1.43]	1.36 [1.28–1.51]			–	**0.000 (0.86)**
	Activity classification	76.7	43.3	76.7	65.6			–	**0.000**
Sorting	MET value	1.72 [1.65–1.95]	1.87 [1.71–2.08]	1.75 [1.67–1.92]	1.80 [1.69–1.89]				–
	Activity classification	16.7	6.7	16.7	13.3				–
Overall	MET value	1.39 [1.29–1.47]	1.53 [1.39–1.71]	1.36 [1.34–1.48]	1.43 [1.36–1.53]	**0.000**			
	Activity Classification	70.8	48.3	70.8	63.3	**0.000**			
		**Workplace Effects**							
Overall	MET value				**0.000**				
	Activity classification				**0.000**				
vs. ActiveChair	MET value	**0.000 (0.69)**	–						
	Activity classification	**0.000**	–						
vs. Standing	MET value	1.000	**0.000 (0.66)**	–					
	Activity classification	1.000	**0.000**	–					

MET value as median with 95% confidence interval, activity classification as % of subjects with MET ≤1.5 (equal to minimum-intensity physical activity classification). Significant p-values marked in bold, effect size given in brackets for significant MET effects (task effects on the right side of the table, workplace effects on the lower side of the table)

The Generalized Estimation Equations used the exchangeable working correlation structure and the robust covariance matrix. A goodness of fit of 38.0 (MET value, linear response) and 306.8 (activity classification, binary logistic response) was observed (QICC value). Abbreviations: Conventional chair (ConvChair), activity-promoting chair (Active Chair), metabolic equivalent (MET)

There was an overall task effect on the MET value and the activity classification as well (Table 2). Sorting caused the highest MET and lowest minPA classification (*p* < 0.001, ES ≥0.86), and Deskwork caused a higher MET and lower minPA classification then Keyboard and Mouse (*p* < 0.001, ES ≥0.40). Despite no difference in the minPA classification, Keyboard caused a significantly higher MET value compared to Mouse (*p* = 0.002, ES = 0.42).

### Workplace and task effects on cpm

There was an overall workplace effect on the waist VA and VM but not the wrist cpm (Table 3). The waist VA and VM cpm were highest for the activity-promoting chair (*p* ≤ 0.007, ES ≥0.34), and lowest for the conventional chair (*p* ≤ 0.003, ES ≥0.63).

**Table 3:** Comparison of the counts-per-minute for the waist and wrist placement for all workplaces and tasks

**Tasks**			**Workplaces**			**Task Effects**			
		ConvChair	ActiveChair	Standing	Overall	Overall	vs. Keyboard	vs. Deskwork	vs. Sorting
Mouse	waist VA	0 [0–0]	29 [4–81]	5 [0–14]	14 [4–35]		1.000	**0.001 (0.59)**	**0.000 (0.85)**
	waist VM	0 [0–1]	457 [268–874]	6 [0–32]	158 [101–301]		0.271	**0.000 (0.29)**	**0.000 (0.75)**
	wrist VM	8 [0–161]	22 [6–74]	51 [6–99]	32 [12–153]		0.114	**0.000 (0.87)**	**0.000 (0.87)**
Keyboard	waist VA	0 [0–0]	17 [4–65]	3 [0–7]	10 [4–33]		–	**0.002 (0.62)**	**0.000 (0.81)**
	waist VM	0 [0–1]	216 [169–438]	4 [0–24]	78 [66–150]		–	**0.000 (0.57)**	**0.000 (0.84)**
	wrist VM	166 [122–314]	174 [128–362]	159 [98–402]	195 [139–344]		–	**0.000 (0.87)**	**0.000 (0.87)**
Deskwork	waist VA	4 [2–9]	78 [43–239]	31 [19–50]	51 [38–94]			–	**0.000 (0.85)**
	waist VM	8 [4–14]	468 [239–657]	44 [25–94]	174 [103–266]			–	**0.000 (0.84)**
	wrist VM	2′427 [1′972–2′620]	2′331 [1′997–2′484]	2′445 [2′137–2′811]	2′393 [2′220–2′629]			–	**0.000 (0.87)**
Sorting	waist VA	221 [141–331]	375 [249–555]	424 [354–567]	356 [289–480]				–
	waist VM	407 [269–520]	929 [662–1′463]	754 [556–809]	707 [585–868]				–
	wrist VM	7′744 [7′058–8′003]	7′664 [7′139–8′281]	7′899 [7′113–8′274]	7′778 [7′048–8′216]				–
Overall	waist VA	59 [36–88]	150 [117–243]	113 [101–176]	120 [93–157]	**0.000**			
	waist VM	109 [69–139]	631 [443–835]	210 [158–239]	321 [246–448]	**0.000**			
	wrist VM	2′625 [2′466–2′806]	2′563 [2′440–2′882]	2′736 [2′481–2′905]	2′658 [2′414–2′866]	**0.000**			
		**Workplace Effects**							
Overall	waist VA				**0.000**				
	waist VM				**0.000**				
	wrist VM				0.742				
vs. ActiveChair	waist VA	**0.003 (0.67)**	–						
	waist VM	**0.000 (0.86)**	–						
	wrist VM	N/A	–						
vs. Standing	waist VA	**0.000 (0.63)**	**0.007 (0.34)**	–					
	waist VM	**0.000 (0.66)**	**0.000 (0.74)**	–					
	wrist VM	N/A	N/A	–					

Counts-per-minute as median with 95% confidence interval. Significant p-values marked in bold, effect sizes given in brackets (task effects on the right side of the table, workplace effects on the lower side of the table)

The Generalised Estimation Equations used the exchangeable working correlation structure and the robust covariance matrix for a negative binomial distribution with log link function. A goodness of fit of 909.9 (waist VA), 798.2 (waist VM), and 647.6 (wrist VM) was observed (QICC values). Abbreviations: Conventional chair (ConvChair), activity-promoting chair (Active Chair), vertical axis (VA), vector magnitude (VM), not applicable (N/A, due to lack of a significant overall effect)

There was an overall task effect on the waist and the wrist cpm. Both placements significantly differed between all tasks (*p* ≤ 0.002, ES ≥0.29), except between Mouse and Keyboard (*p* ≥ 0.114).

### Concurrent validity to detect SB, minPA, and sitting

**Fig. 2:**
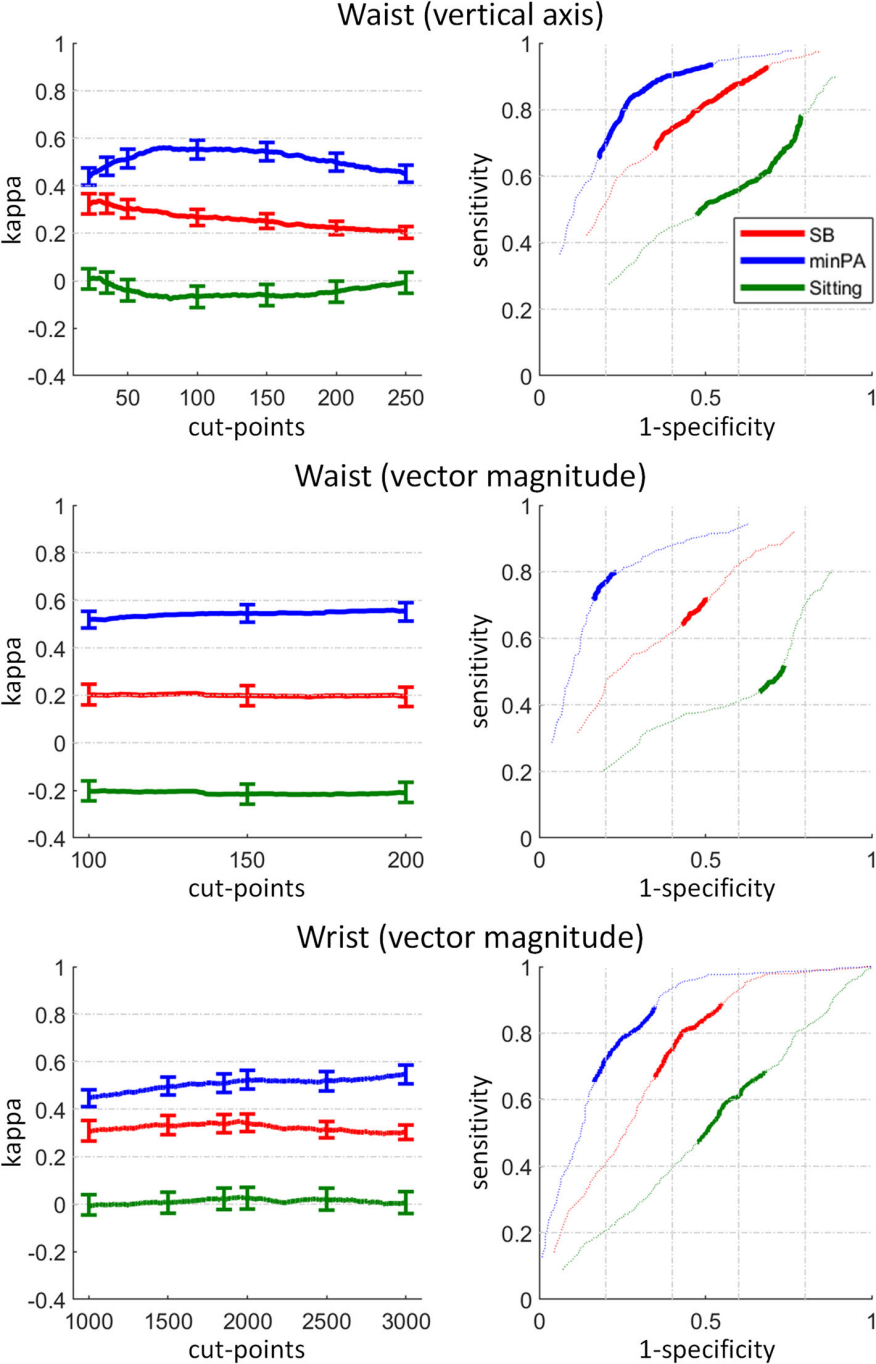
Concurrent validity to detect sedentary behaviour (SB), minimal-intensity physical activity (minPA), and sitting. Kappa (left) error bars denote the 95% confidence interval of commonly used counts-per-minute (cpm) cut-points. The dotted lines show the ROC curves (right) for lower (down to 0) and higher cut-points (up to 500 and 750 for waist VA and VM and 15′000 for wrist VM). Definition of SB, minPA, and sitting is given in Table 1

All sensors reached a fair agreement with the reference criteria to detect SB (kappa: 0.21–0.35), a moderate agreement to detect minPA (0.52–0.56), and a poor to slight agreement to detect sitting (-0.20–0.03, Fig. 2). The ROC curves showed for both placements the same pattern as kappa, with the detection of minPA having the highest sensitivities and specificities and sitting the lowest, rarely above the 45° line.

### Concurrent validity to detect minPA, separated by workplace and task

**Fig. 3:**
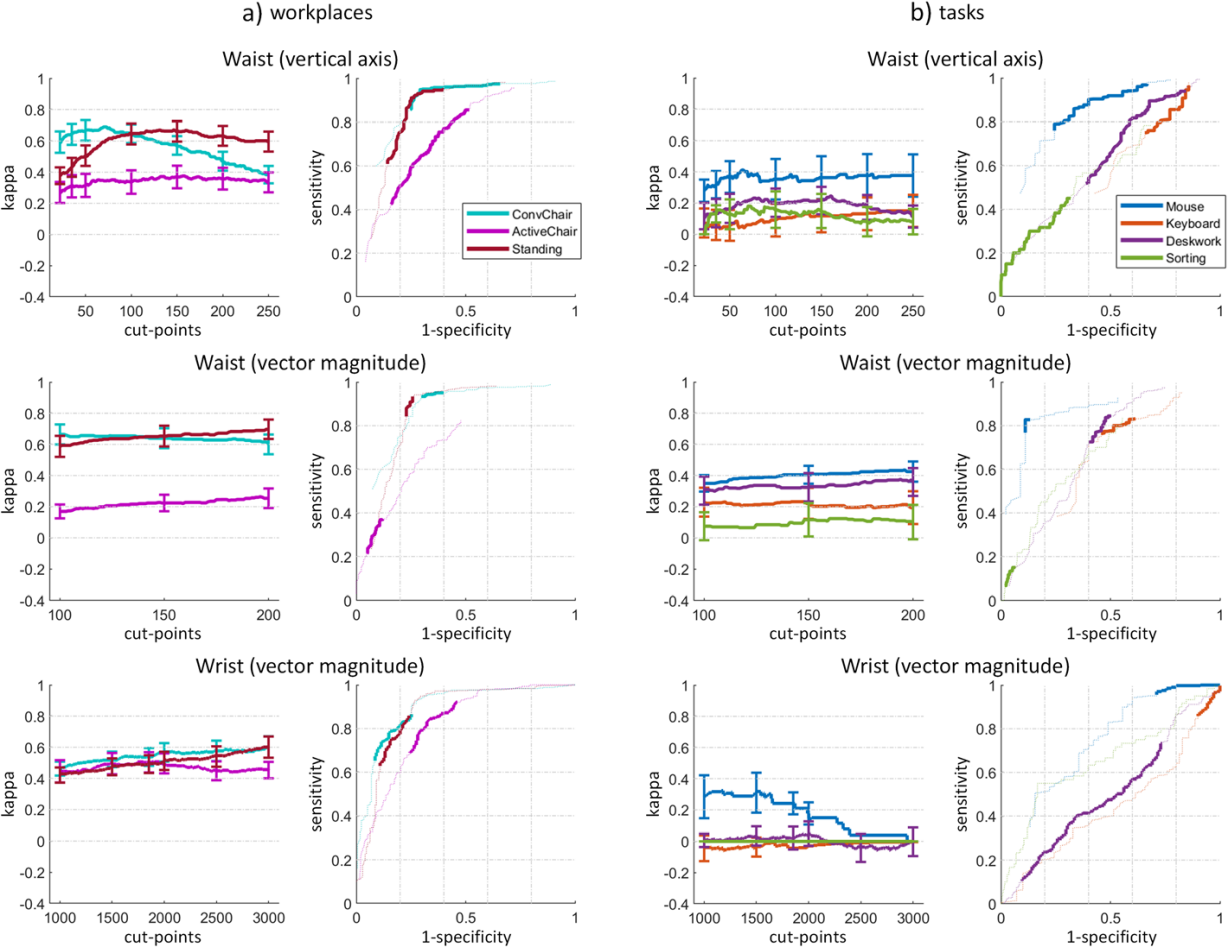
Concurrent validity to detect minimal-intensity physical activity (minPA) separated by workplace (**a**) and task (**b**). Kappa (left) error bars denote the 95% confidence interval of commonly used counts-per-minute (cpm) cut-points. The dotted lines show the ROC curves (right) for lower (down to 0) and higher cut-points (up to 500 and 750 for waist VA and VM and 15′000 for wrist VM). Definition of minPA is given in Table 1

The minPA detection for each workplace (Fig. 3a) showed for the waist VA and VM a substantial agreement with the reference criterion for the conventional chair and the standing desk (kappa: 0.66–0.70), and a fair agreement for the activity-promoting chair (0.26–0.38). The wrist sensor reached for all workplaces a moderate agreement with the reference criterion (0.51–0.60). For both placements, the ROC curves for the conventional chair and the standing desk were similar but slightly shifted (conventional chair with higher sensitivity and lower specificity), while the curve for the activity-promoting chair was lower.

The minPA detection for each task (Fig. 3b) showed a less clear pattern, except that the Mouse reached the highest agreement with the reference criterion for both placements, moderate for the waist (0.41–0.44) and fair for the wrist (0.32). In line, the Mouse reached the highest sensitivities and specificities in the ROC curves.

### Discriminant validity for the behaviour, activity, and posture classification

**Fig. 4:**
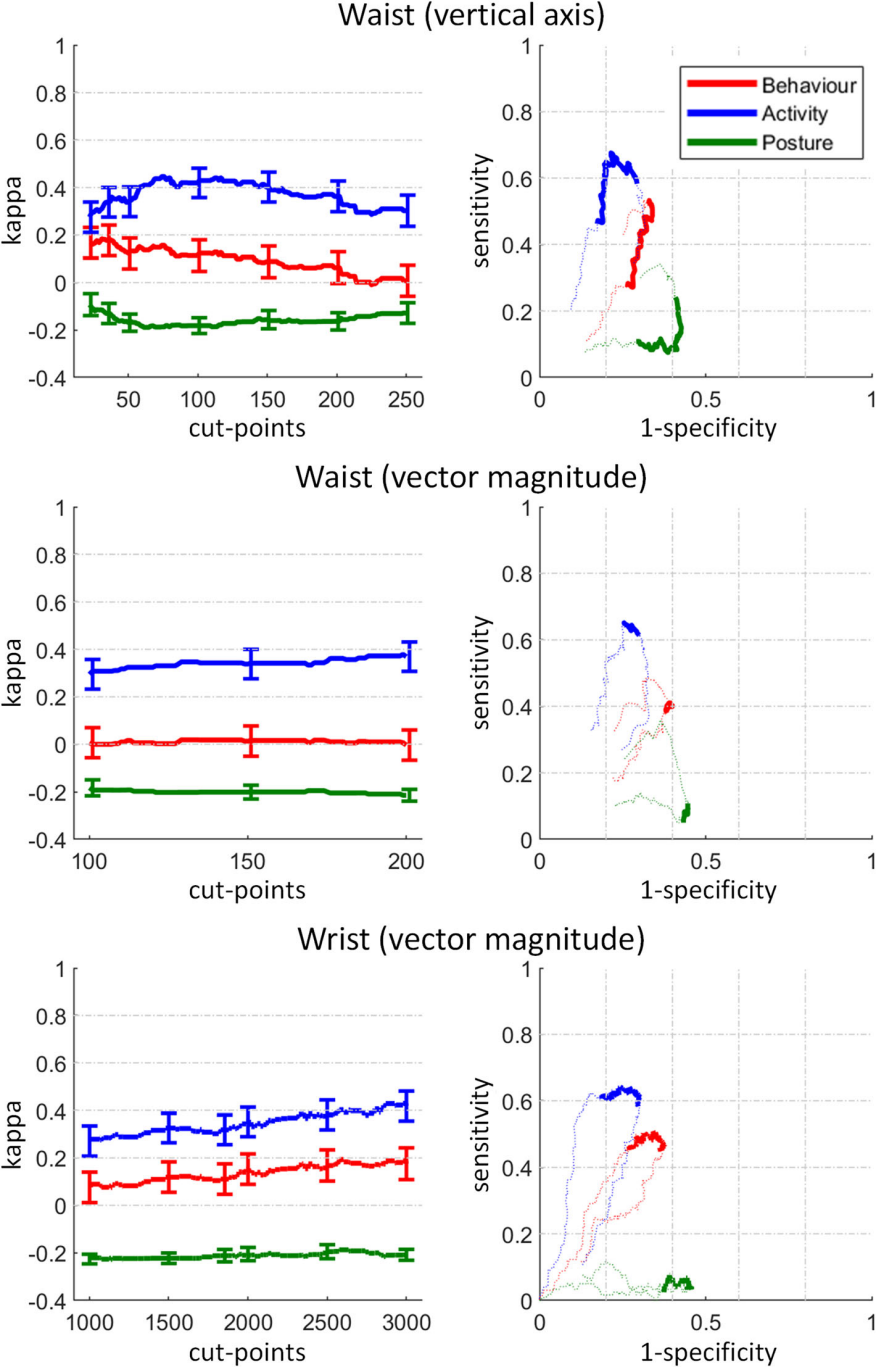
Discriminant validity for the behaviour, activity, and posture classification. Kappa (left) error bars denote the 95% confidence interval of commonly used counts-per-minute (cpm) cut-points. The dotted lines show the ROC curves (right) for lower (down to 0) and higher cut-points (up to 500 and 750 for waist VA and VM and 15′000 for wrist VM). Definition of behaviour, activity level, and posture is given in Table 1

All sensors reached a slight agreement with the reference criteria to discriminate the behaviour (SB and non-SB, kappa: 0.02–0.19, Fig. 4), a fair to moderate agreement to discriminate the activity level (minPA and LPA, 0.38–0.45), and a poor agreement to discriminate posture (sitting and standing, -0.19 – -0.09). The ROC curves in Fig. 4 do not proceed from the origin (0% sensitivity, 100% specificity) to the most upper right point (100% sensitivity, 0% specificity) since the sensitivity/specificity to discriminate between real differences does not continuously increase/decrease with increasing cut-point.

### Discriminant validity for the behaviour, activity, and posture classification, separated by workplace and task effects

For workplace effects, the waist VA and VM showed a slight and poor agreement with the reference criteria to discriminate the behaviour (SB and non-SB, kappa: 0.10 and -0.20), a fair agreement to discriminate the activity level (minPA and LPA, 0.22–0.24), and a poor agreement to discriminate posture (sitting and standing, -0.44 – -0.10). The wrist reached for all classifications a slight agreement with the reference criteria (0.08–0.13). While the waist reached a sensitivity of up to 72% (VM, activity level), the wrist never exceeded a sensitivity of 11%.

For task effects on the activity level, both placements showed a moderate agreement with the reference criteria (0.42–0.48). The sensitivity reached for both placements 75% (waist) and 79% (wrist).

## Discussion

This study analysed the concurrent and discriminant validity of commonly used waist and wrist cpm cut-points while performing four office tasks (Mouse, Keyboard, Deskwork, Sorting) at three workplaces (conventional chair, activity-promoting chair, and standing). The concurrent validity analysed the ActiGraph’s validity to detect SB (≤1.5 MET while sitting), minPA (≤1.5 MET regardless of body posture), and sitting. The discriminant validity analysed the ActiGraph’s validity to separate SB from non-SB (behaviour classification), to separate minPA from LPA (activity classification), and to separate sitting from standing (posture classification).

In general, the validity was higher for the minPA than for the SB and sitting detection, and higher for the activity than the behaviour and posture discrimination. Accordingly, the ActiGraph cpm cut-points represent a measure for activity, and not for SB or posture. Furthermore, the validity was higher to detect task effects than to detect workplace effects. Caution is warranted when analysing workplace effects on the activity level with ActiGraph cpm cut-points.

### Concurrent validity to detect SB, minPA, and sitting

No matter which sensor placement nor axis was analysed, there was an overall fair agreement with the reference criteria to detect SB in the study population (Fig. 2). However, when the same cut-points are interpreted as detection of minPA, the agreement was higher (moderate) and the ROC curves showed higher sensitivities and specificities. For the conventional chair and the standing desk, there was even a substantial validity to detect minPA with the waist placement. This indicates that the cpm cut-points investigated in this study measure minPA. However, the workplace analysis in Fig. 3a shows a serious limitation of the waist cut-points with respect to posture. For the VA, the cut-point with highest kappa to detect minPA was substantially higher for the standing desk (around 150 cpm) than for the conventional chair (around 75 cpm). If using the same cut-point to detect minPA for both workplaces (e.g. 100 cpm with a kappa of 0.65 for both workplaces), the detection of minPA has a higher sensitivity and lower specificity for sitting than standing (see ROC curve in Fig. 3a and separate sensitivity and specificity in Additional file 3 - Fig. 2). Thus, standing will be likely classified as more active than sitting even when there is no true difference in the activity level as in the present study. The same observation was also made for the waist VM. This limitation is likely caused by the fixed pelvis height in sitting, which causes fewer cpm compared to standing. A similar observation has repeatedly been reported for cycling [50, 51]. Based on these results, the waist VA and VM should only be used in combination with a posture-based sensor and posture-specific cut-points in case the activity level at a sit-stand desk is analysed. A moderate valid alternative to detect minPA would be to use the wrist VM with similar sensitivities and specificities for the conventional chair and the standing desk.

Although developed to detect physical activity, the ActiGraph was repeatedly calibrated to detect sitting [29, 34–36]. For example, Koster and colleagues [36] reported only slightly different balanced sensitivities and specificities to detect sitting as this study observed to detect minPA (Koster vs. this study): 75.9% vs. 77.1% (waist-worn, VA, 100 cpm), 83.7% vs. 78.1% (waist-worn, VM, 200 cpm), and 79.1% vs. 76.4% (wrist-worn, VM, 1853 cpm). For the sitting detection, this study observed for all cut-points sensitivities and specificities very close to or even below the 45° line in the ROC curve with mostly negative kappa, indicating a similar or lower validity than what would be expected by chance. The large difference is likely a result of the measurement setting. This study recorded exactly the same tasks in sitting and standing, and the observed sensitivities and specificities depended only on body posture. In contrast, the cited studies recorded the natural behaviour in a free-living setting where a substantial agreement between minPA and sitting exists [4, 52]. Matthews and colleagues [4] reported an average minPA time of 9.7 h a day (ActiGraph waist, VA, 100 cpm), and an average sitting time of 9.8 h a day (activPAL). However, the relationship between the minPA and sitting time varies depending on the study sample, which is why different studies recommend different cut-points to get the most similar minPA and sitting time [29, 34–36]. Clarke-Cornwell and colleagues [34] noticed such a variation even within one sample in relation to day (weekday vs weekends) and task (working vs non-working). Based on our results, we clearly advise against interpreting the ActiGraph measured time as sitting. If the sitting time is of interest, a posture-based device should be employed. If the minPA time is of interest, an activity-based device should be employed. Unless both devices are combined, one should not interpret the results as if SB has been measured. Only when the different constructs are correctly applied, it will get possible to investigate whether minPA, sitting, or the combination thereof (SB) causes the detrimental health effects currently associated with SB. While this clear distinction might be less important for epidemiological studies due to the substantial agreement of minPA and sitting time in free-living, it is all the more important when studying the effects of workplace interventions. These are typically aimed to break up either minPA (e.g. activity-promoting chairs) or sitting (e.g. sit-stand desks), but rarely both (e.g. treadmill desks). For example, if it turns out that the health effects currently associated to SB are in fact caused by minPA but not by sitting, the recommendation for using standing workplaces could be questioned from the sedentary research perspective. Furthermore, if the measured times are interpreted on an individual level to detect daily behaviour patterns or prolonged sitting bouts [53], the simplification of equating minPA with sitting holds no longer true. In line with Matthews et al. [4], we noticed in a recent study a very small bias (− 7 min per day) between minPA (ActiGraph, waist-worn, VA, 100 cpm) and sitting (activPAL), but a very large bias (− 105 min per day) between the two sensors when looking at the time spent in bouts ≥10 min [54]. A similar underestimation of prolonged sitting was found in other studies [52, 55], indicating that prolonged sitting (activPAL) contains some LPA minutes (ActiGraph) breaking up SB. In this respect, we consider it important to combine the activPAL to measure sitting with the ActiGraph to measure minPA in order to uncover the true activity level of free-living sitting and standing, inside and outside the office workplace. This combination would further allow to use posture-specific cut-points for the activity classification, to measure SB in line with its definition [1], and ultimately to fuse the physical behaviour measurements of occupational and public health.

### Discriminant validity for the behaviour, activity, and posture classification

The validity to discriminate activity level into minPA and LPA was substantially higher than the validity to discriminate behaviour into SB and non-SB and to discriminate posture into sitting and standing. Accordingly, differences in cpm cut-point classifications should be interpreted as differences in the activity level, and not as differences in the behaviour or posture classification.

### Activity-promoting chair

To inspect the cut-points’ ability to detect the effect of an active workplace intervention, the study combined an activity-promoting office chair with an oral prompting. This combination had a strong effect on the activity level compared to the conventional chair and the standing desk. It significantly increased the participants MET by around 13%, thereby reducing the minPA time and thus SB by around 32%.

In line, the average cpm for the waist VA and VM significantly differed between the activity-promoting and conventional chair, while there was no workplace effect for the wrist VM (Table 3). This observation can be explained by the investigated chair: The activity-promoting chair reduced the time in minPA by continuous lateral pelvis movements increasing the waist but not the wrist cpm. Accordingly, the waist reached a higher, but still only a fair validity to discriminate the activity level. It is therefore questionable whether the waist placement detects a true activity change induced by a seated active workplace intervention in a future field study. This is all the more remarkable as the activity-promoting chair directly affected the movement pattern of the pelvis. We assume that the conversion from the raw acceleration to the cpm is not sensitive enough to detect an increased activity caused by a continuous, impact-free pelvis motion with a cut-point approach, and would expect even lower validities for an active workplace intervention not affecting the pelvis motion (e.g. cycling desk). Consequently, a seated, impact-free active workplace intervention should not be analysed with a waist or wrist cut-point.

### Standing desk

Due to the prescribed posture, the standing desk eliminated SB completely, but there was no effect on the MET value nor on the activity classification. This observation is in line with [56, 57] but stays in contrast to [58, 59].

Despite the same activity level, there was a strong workplace effect on the waist VA and VM cpm (ES ≥0.63), both having significantly higher cpm for the standing desk than the conventional chair. In a study investigating the effect of a sit-stand-desk on the time spent in minPA with a waist-worn sensor (e.g. [60]), this might wrongly result in standing to be considered more active than sitting. This observation underlines our recommendation to combine the waist placement with a posture-based sensor and apply posture-specific cut-points if participants use a sit-stand desk, and stays in contrast to the Cochrane review on workplace interventions that considered the ActiGraph waist as valid to evaluate the effect of sit-stand desks on SB [18]. The wrist sensor, on the other hand, had no different cpm value between conventional sitting and the standing desk, and detected minPA with a moderate agreement for all workplaces. Thus, if the waist sensor is not combined with a posture-based sensor, the wrist should be the placement of choice to analyse the effect of sit-stand desks on the activity level.

In line to the poor concurrent validity to detect sitting, the validity to discriminate sitting and standing was poor and below what would be expected by chance for all cut-points. We therefore conclude, in accordance with previous research [61], that the discrimination of posture into sitting and standing with cut-points is not valid.

### Task effects

**Fig. 5:**
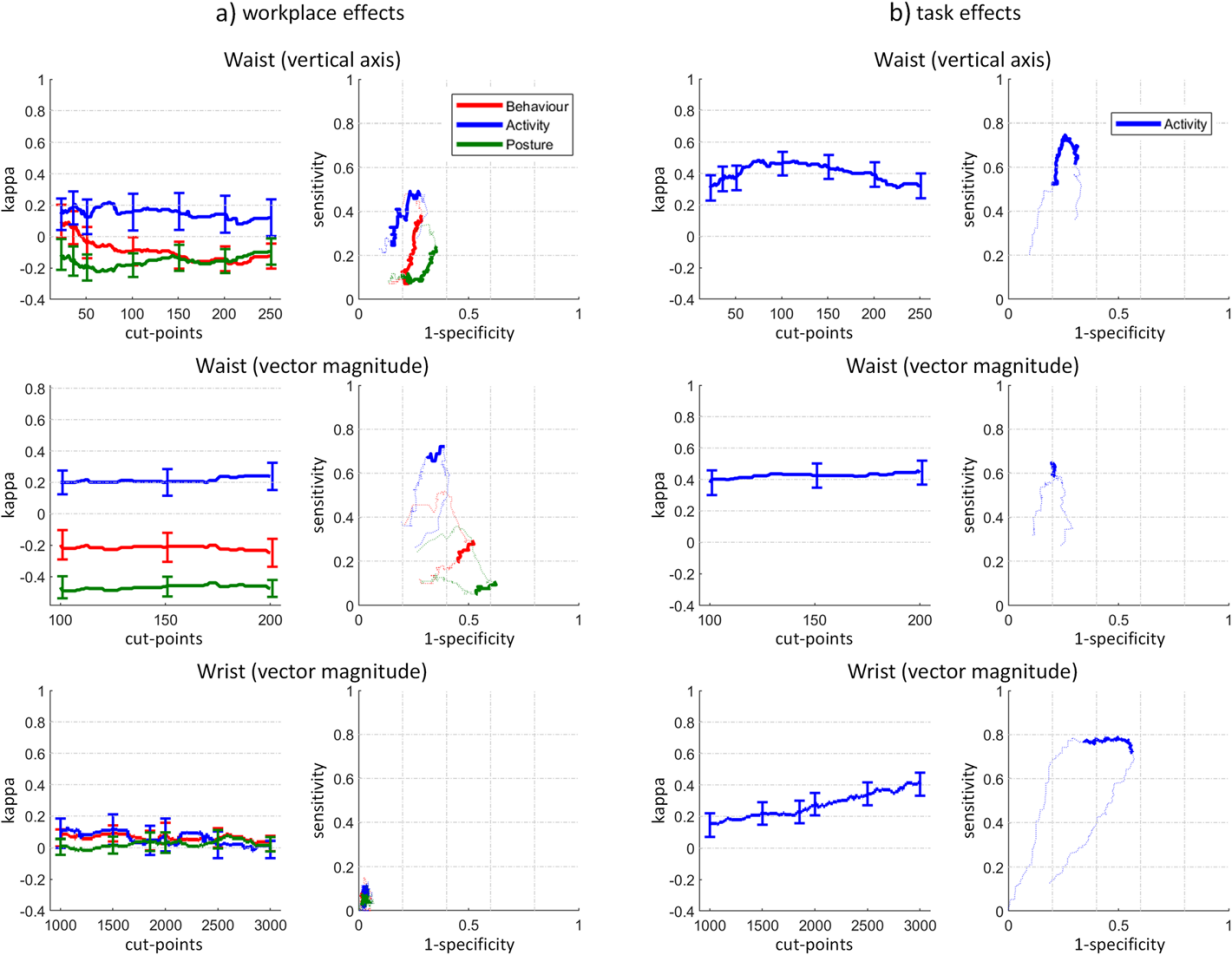
Discriminant validity for the behaviour, activity, and posture classification, separated by workplace and task effects. Kappa (left) error bars denote the 95% confidence interval of commonly used counts-per-minute (cpm) cut-points. The dotted lines show the ROC curves (right) for lower (down to 0) and higher cut-points (up to 500 and 750 for waist VA and VM and 15′000 for wrist VM). Note that the workplace effect for waist (VM) shows a lower y-axis range for kappa. Since task effects never changed posture, only the activity classification is shown for task effects. Definition of behaviour, activity level, and posture is given in Table 1

Research repeatedly observed that the activity level is more influenced by the office task than the workplace [12, 62]. Although this study noticed a strong effect of the activity-promoting chair, it noticed an even stronger effect when changing from a computer-assisted task (Mouse, Keyboard) to a non-computer-assisted task (Sorting, Table 2). The average cpm for all tasks were significantly different, except between Mouse and Keyboard, which had a different MET but no different activity level. The validity to discriminate the activity level caused by task effects was moderate for the waist and fair for the wrist, and all cut-points performed better to discriminate between task effects than workplace effects (Fig. 5). In fact, the waist cut-points are moderate valid to detect task effects on the activity level.

### Strengths and limitations

The present study measured an office worker sample while performing office work, and the findings might not be transferrable to situations outside the office. On the other hand, the majority of the population works in the office sector, and office work is the single largest contributor to SB in the population. Office work is therefore a highly relevant behaviour, even for population-based epidemiological studies. The included sample contained office workers in a wide range of age (23–57 years), weight (55–100 kg), height (1.55–1.91 m) and BMI (19.5–33.0 kg/m^2^), with eight overweight and one obese, but 2/3 were below 40 years. Most of the participants reported to stand less than 5 h a week at their desk, four between 5 to 10 and 10 to 15 h, respectively, and one > 15 h. The average office worker spends around 64–82% of the working time sedentary, while our sample reported to sit for 78% of their working time at an office desk. Thus, we consider our sample to be representative for a general office worker population despite its slightly young age.

This study showed that the three included workplaces were actually different from each other, and both the activity-promoting chair (reducing minPA) and the standing desk (eliminating sitting) reduced SB. To analyse the sensitivity of the observed effects, the entire inferential statistic was repeated with traditional methods (Friedman test), and exactly the same effects as with the Generalized Estimation Equations were observed. However, whether the activity-promoting office chair in combination with oral prompting is an effective intervention to break up SB at office workplaces remains subject to future field studies. Due to the study method with four office tasks performed at each of the three workplaces, the results of the posture analysis are solely caused by body posture, unaffected by the activity level. This is why the presented validities stay in contrast to those reported in field studies with a substantial agreement between sitting and minPA.

Another strength of this study is the inclusion of both a waist- and wrist-worn ActiGraph, and the analysis of VA and VM for the waist sensor. Furthermore, the study used both kappa and ROC curves and studied the concurrent and the discriminant validity, for the low-frequency-extension and the normal filtering (see Additional file 1). We are aware that kappa is affected by the prevalence- and bias-index [63]. The interested reader therefore finds in Additional files 1 and 3 the prevalence-adjusted-bias-adjusted-kappa. However, we consider the kappa to reflect the true situation penalized for prevalence and bias, while the prevalence-adjusted-bias-adjusted-kappa relates to a hypothetical situation with balanced behaviours [63]. With respect to office work, the prominent minPA is a true challenge for each measurement system.

The data of the indirect calorimeter were processed on a task-by-task level, while the ActiGraph data were processed on the typical minute-by-minute level. In case the true activity level was for one task LPA for 1 min and minPA for 4 min, our reference criterion classified the entire 5 min as minPA. It therefore might be that the validity of the ActiGraph cut-points is marginally higher than reported due to the task-by-task processing for the reference criterion. However, no substantial deviations in the individual minute analysis were found (Additional file 2). In this regard, it is also important to mention that the lab-based nature of the present study with only a selection of office tasks is a further limitation. The participants of this study performed each task for 5 min to ensure the detection of a steady-state MET, while in free-living office workers might change from one task to another and from one workplace to another several times a minute. However, office workers are known to spend most of their working time sedentary, and accumulate their sedentary time predominantly in long bouts with rare posture changes [9, 10]. Furthermore, this study classified all tasks into minPA and LPA, although one subject exceeded once the upper MET threshold for LPA (3.0 MET) and should have been classified as moderately physically active. To separate the activity level in minPA and LPA, the study used a threshold of 1.5 MET [1, 64]. This threshold is the reason why the Mouse and Keyboard have a significantly different MET but a similar activity classification. Although widely accepted, it is important to note that the 1.5 MET threshold is arbitrary and there is no health-relevant evidence supporting the threshold. We therefore reported the MET along with the activity level. An increase from 1.35 to 1.45 MET might have similar health effects as an increase from 1.45 to 1.55 MET.

## Conclusion

This study showed that the ActiGraph cpm for waist and wrist placement is, independently of the chosen cut-point, a measure for the activity level and not for SB or sitting. The cpm cut-points performed generally better to discriminate the activity level caused by task effects than workplace effects. Waist cut-points were most valid to measure the activity level in conventional seated office work, but they showed severe limitations for sit-stand desks. None of the placements were able to detect the increased activity on the activity-promoting chair. Caution is therefore warranted when analysing workplace interventions such as activity-promoting office chairs and standing desks with ActiGraph waist and wrist cut-points.

## Supplementary Information

**Additional file 1.** Normal Filtered Data, showing the data presented in Table 3 and Figs. 2, 3, 4 and 5 for the normal filtered instead of the low-frequency-extension filtered data, including the prevalence-adjusted-bias-adjusted-kappa and the sensitivity and specificity.

**Additional file 2.** All minutes versus single minutes, comparing the data of the all minutes approach as presented in Figs. 2 and 4 with the analysis of each single minute.

**Additional file 3.** Prevalence-adjusted-bias-adjusted-kappa and sensitivity and specificity, shown for each figure in the manuscript.

## Data Availability

The datasets analysed during the current study are available from the corresponding author on reasonable request.

## Abbreviations

SB: Sedentary Behaviour
MET: Metabolic Equivalent
minPA: Minimal-intensity physical activity
LPA: Light-intensity physical activity
cpm: Counts-per-minute
VA: Vertical Axis
VM: Vector Magnitude
ES: Effect Size
