# Supplementary material for: Concurrent and discriminant validity of ActiGraph waist and wrist cut-points to measure sedentary behaviour, activity level, and posture in office work
Source: BMC Public Health. 2021 Feb 12;21:345. doi: 10.1186/s12889-021-10387-7 (PMC7881682; doi:10.1186/s12889-021-10387-7)

Additional File 1 - Table 1: Counts-per-minute for the waist and wrist placement for all workplaces and tasks.

		**Workplaces**				**Task Effects**			
**Tasks**		ConvChair	ActiveChair	Standing	Overall	Overall	vs. Keyboard	vs. Deskwork	vs. Sorting
Mouse	waist VA	0 [0 - 0]	0 [0 - 5]	0 [0 - 5]	2 [0 - 5]		1.000	0.116	**0.000 (0.85)**
	waist VM	0 [0 - 0]	133 [25 - 500]	0 [0 - 15]	54 [15 - 174]		0.149	**0.048 (0.18)**	**0.000 (0.73)**
	wrist VM	6 [0 - 141]	1 [0 - 31]	32 [3 - 69]	19 [6 - 128]		0.596	**0.000 (0.87)**	**0.000 (0.87)**
Keyboard	waist VA	0 [0 - 0]	0 [0 - 2]	0 [0 - 4]	1 [0 - 5]		-	0.218	**0.000 (0.82)**
	waist VM	0 [0 - 0]	54 [16 - 211]	0 [0 - 14]	25 [9 - 71]		-	**0.000 (0.46)**	**0.000 (0.84)**
	wrist VM	84 [63 - 182]	82 [36 - 188]	73 [37 - 278]	104 [66 - 227]		-	**0.000 (0.87)**	**0.000 (0.87)**
Deskwork	waist VA	0 [0 - 0]	21 [3 - 86]	13 [4 - 23]	22 [8 - 44]			-	**0.000 (0.86)**
	waist VM	0 [0 - 0]	214 [78 - 419]	18 [8 - 42]	88 [38 - 156]			-	**0.000 (0.82)**
	wrist VM	2'307 [1'819 - 2'515]	2'187 [1'860 - 2'377]	2'339 [1'993 - 2'653]	2'273 [2'089 - 2'490]			-	**0.000 (0.87)**
Sorting	waist VA	151 [88 - 245]	219 [140 - 378]	339 [266 - 467]	246 [201 - 345]				-
	waist VM	290 [184 - 410]	676 [350 - 1’236]	634 [463 - 688]	546 [397 - 713]				-
	wrist VM	7'581 [6'884 - 7'845]	7'512 [6'966 - 8'133]	7'743 [6'927 - 8'131]	7'624 [6'886 - 8'064]				-
Overall	waist VA	38 [22 - 67]	69 [41 - 123]	87 [72 - 133]	70 [51 - 107]	**0.000**			
	waist VM	74 [46 - 103]	404 [184 - 587]	168 [122 - 195]	219 [131 - 329]	**0.000**			
	wrist VM	2'552 [2'374 - 2'725]	2'477 [2'329 - 2'762]	2'633 [2'393 - 2'773]	2'561 [2'301 - 2'777]	**0.000**			
			**Workplace Effects**						
Overall	waist VA				**0.000**				
	waist VM				**0.000**				
	wrist VM				0.900				
vs. ActiveChair	waist VA	0.066	-						
	waist VM	**0.000 (0.79)**	-						
	wrist VM	N/A	-						
vs. Standing	waist VA	**0.000 (0.54)**	0.119	-					
	waist VM	**0.000 (0.61)**	**0.001 (0.56)**	-					
	wrist VM	N/A	N/A	-					

Indicated are the normal filtered median counts-per-minute with 95% confidence interval for waist vertical axis (VA) and vector magnitude (VM), as well as wrist VM. Significant p-values marked in bold. The non-parametric effect size is given in brackets for significant effects. The Generalised Estimation Equations used the exchangeable working correlation structure and the robust covariance matrix for a negative binomial distribution with log link function. A goodness of fit of 1296.4 (waist VA), 1123.0 (waist VM), and 773.9 (wrist VM) was observed (QICC values). N/A: not applicable due to lack of significant overall effect. This Table corresponds to Table 2 in the manuscript but for normal filtered instead of low-frequency-extension filtered data.

Additional File 1 - Figure 1: Kappa, Prevalence-adjusted-bias-adjusted-kappa (PABAK), bias and prevalence index, as well as sensitivity, specificity, and ROC curve to detect sedentary behaviour (SB), minimal-intensity physical activity (minPA), and sitting. Normal filtered data shown for the waist vertical axis (VA), vector magnitude (VM) and wrist VM. The Kappa and PABAK error bars denote the 95% confidence interval of commonly used counts-per-minute (cpm) cut-points (cut-point indicated on x-axis, for waist VA and wrist VM additionally for 22 and 35 cpm and 1’853 cpm, respectively). The dotted lines show the ROC for lower (down to 0) and higher cut-points (up to 500 and 750 for waist VA and VM and 15’000 for wrist VM). Figure corresponds to Figure 2 in the manuscript but for normal filtered instead of low-frequency-extension filtered data.

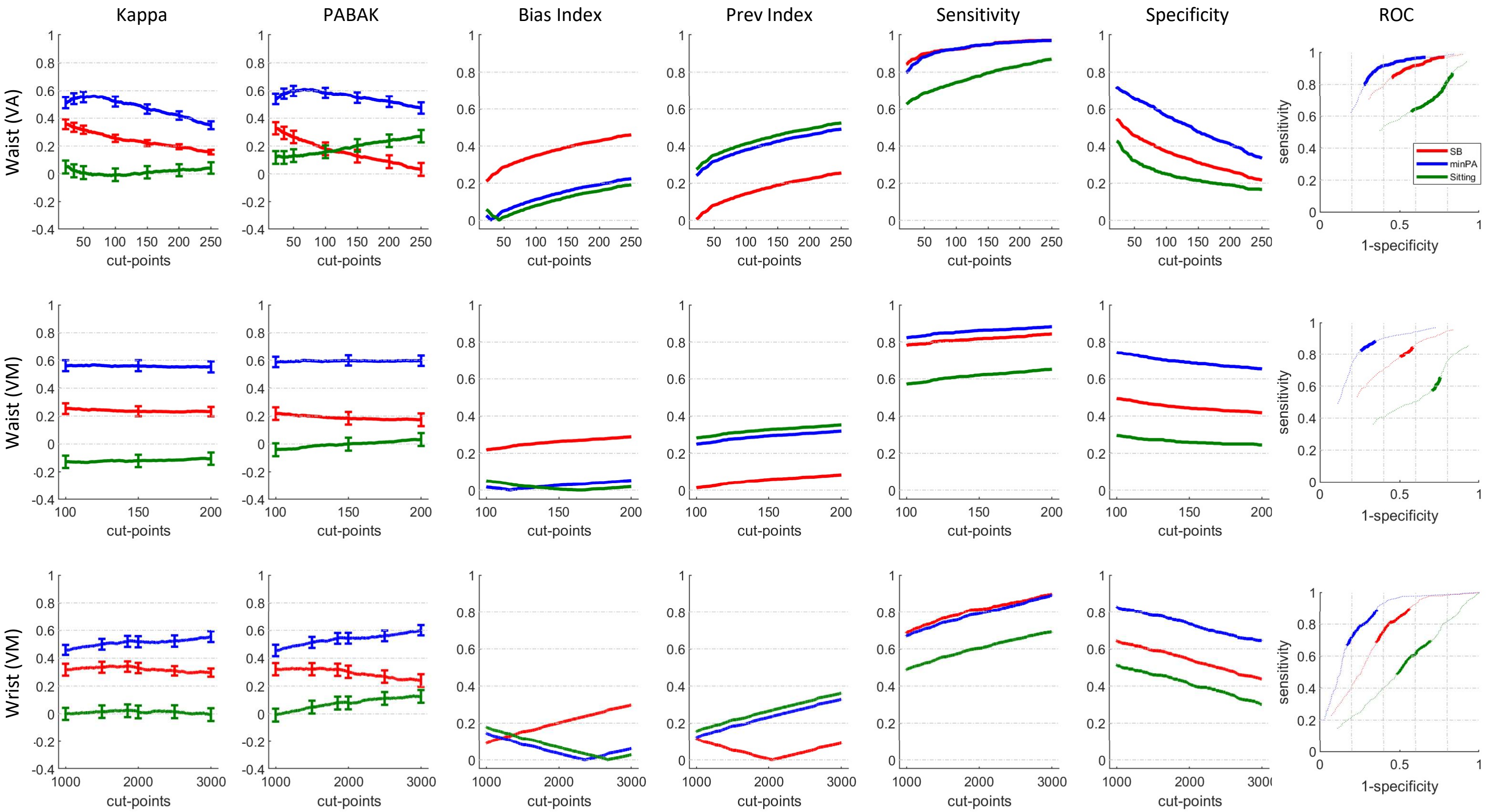

Additional File 1 - Figure 2: Kappa, Prevalence-adjusted-bias-adjusted-kappa (PABAK), bias and prevalence index, as well as sensitivity, specificity, and ROC curve to detect minimal-intensity physical activity (minPA) for each workplace. Normal filtered data shown for the waist vertical axis (VA), vector magnitude (VM) and wrist VM. The Kappa and PABAK error bars denote the 95% confidence interval of commonly used counts-per-minute (cpm) cut-points (cut-point indicated on x-axis, for waist VA and wrist VM additionally for 22 and 35 cpm and 1’853 cpm, respectively). The dotted lines show the ROC for lower (down to 0) and higher cut-points (up to 500 and 750 for waist VA and VM and 15’000 for wrist VM). Figure corresponds to Figure 3a in the manuscript but for normal filtered instead of low-frequency-extension filtered data.

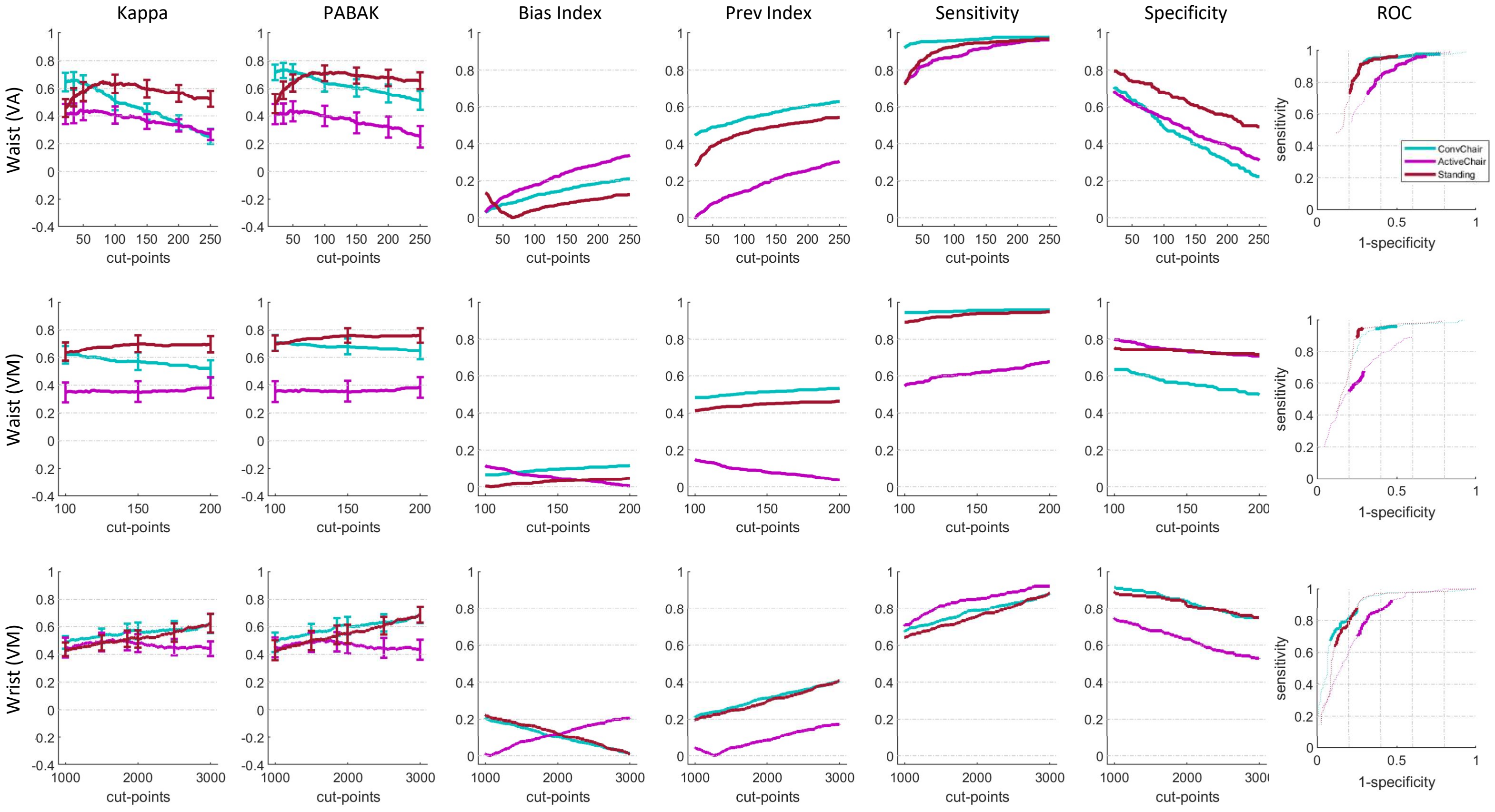

Additional File 1 - Figure 3: Kappa, Prevalence-adjusted-bias-adjusted-kappa (PABAK), bias and prevalence index, as well as sensitivity, specificity, and ROC curve to detect minimal-intensity physical activity (minPA) for each task. Normal filtered data shown for the waist vertical axis (VA), vector magnitude (VM) and wrist VM. The Kappa and PABAK error bars denote the 95% confidence interval of commonly used counts-per-minute (cpm) cut-points (cut-point indicated on x-axis, for waist VA and wrist VM additionally for 22 and 35 cpm and 1'853 cpm, respectively). The dotted lines show the ROC for lower (down to 0) and higher cut-points (up to 500 and 750 for waist VA and VM and 15'000 for wrist VM). Figure corresponds to Figure 3b in the manuscript but for normal filtered instead of low-frequency-extension filtered data.

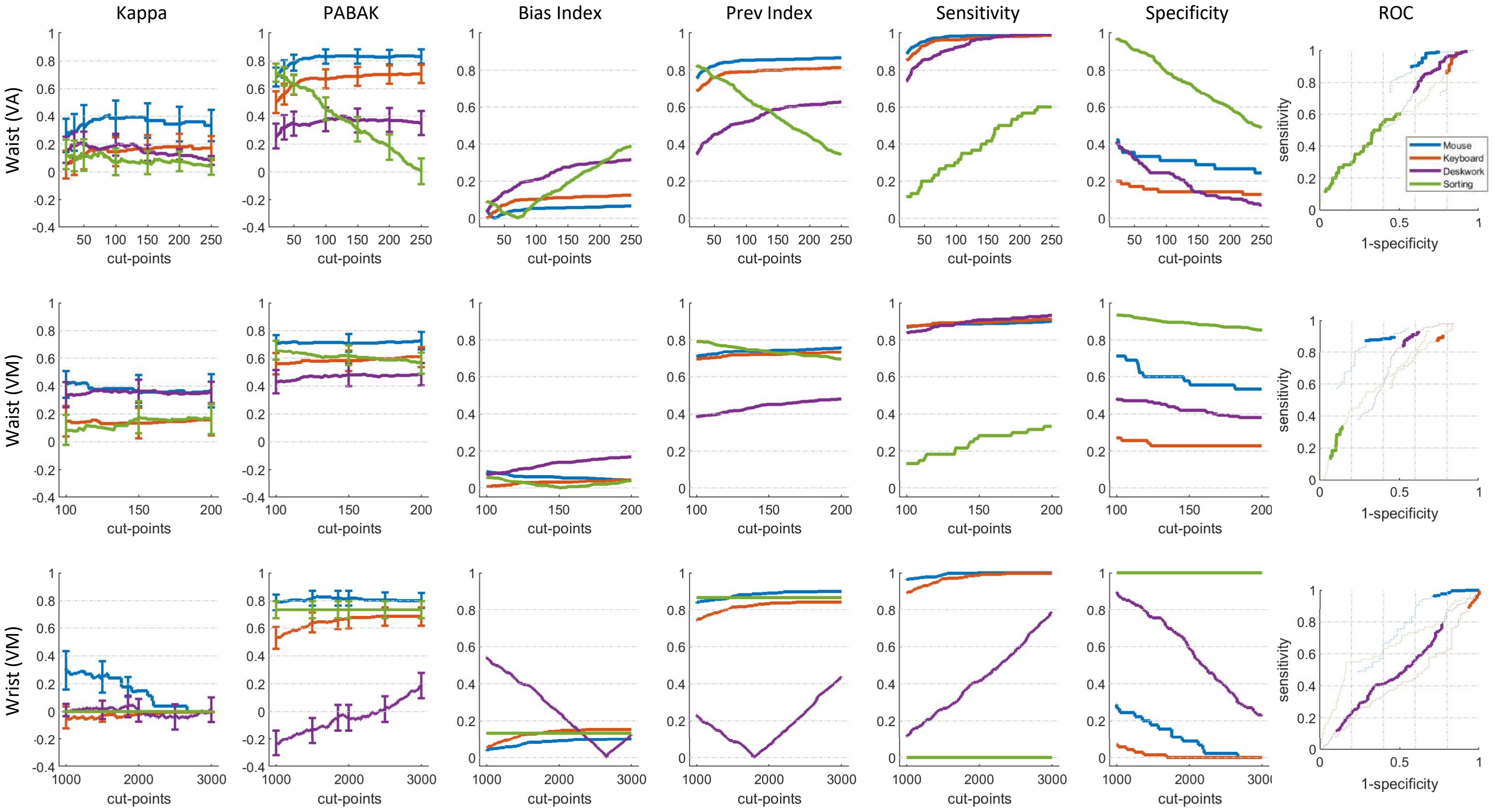

Additional File 1 - Figure 4: Kappa, Prevalence-adjusted-bias-adjusted-kappa (PABAK), bias and prevalence index, as well as sensitivity, specificity, and ROC curve to discriminate sedentary behaviour (SB), activity, and posture. Normal filtered data shown for the waist vertical axis (VA), vector magnitude (VM) and wrist VM. The Kappa and PABAK error bars denote the 95% confidence interval of commonly used counts-per-minute (cpm) cut-points (cut-point indicated on x-axis, for waist VA and wrist VM additionally for 22 and 35 cpm and 1'853 cpm, respectively). The dotted lines show the ROC for lower (down to 0) and higher cut-points (up to 500 and 750 for waist VA and VM and 15'000 for wrist VM). Figure corresponds to Figure 4 in the manuscript but for normal filtered instead of low-frequency-extension filtered data.

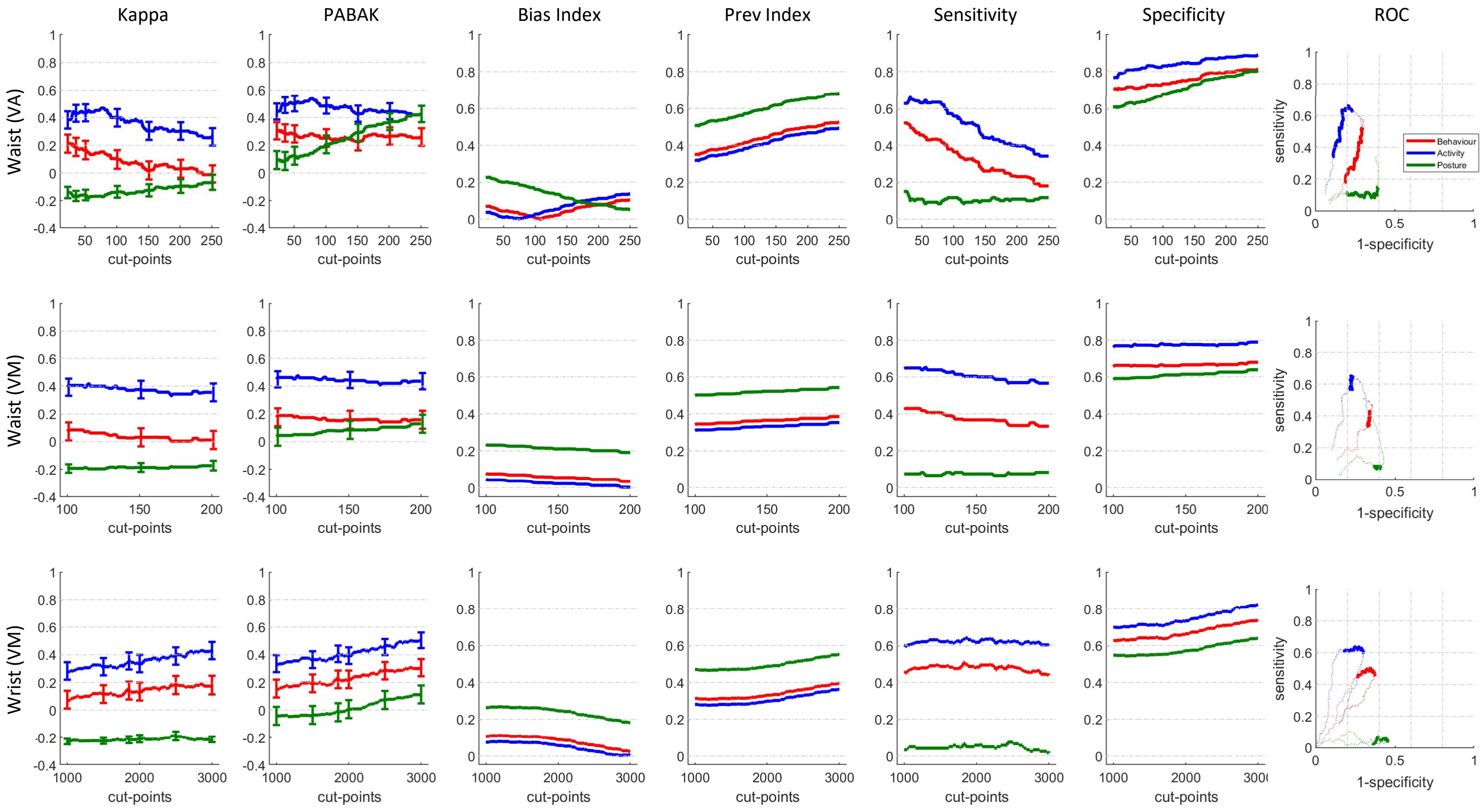

Additional File 1 - Figure 5: Kappa, Prevalence-adjusted-bias-adjusted-kappa (PABAK), bias and prevalence index, as well as sensitivity, specificity, and ROC curve to discriminate workplace effects on sedentary behaviour (SB), activity, and posture. Normal filtered data shown for the waist vertical axis (VA), vector magnitude (VM) and wrist VM. The Kappa and PABAK error bars denote the 95% confidence interval of commonly used counts-per-minute (cpm) cut-points (cut-point indicated on x-axis, for waist VA and wrist VM additionally for 22 and 35 cpm and 1’853 cpm, respectively). The dotted lines show the ROC for lower (down to 0) and higher cut-points (up to 500 and 750 for waist VA and VM and 15’000 for wrist VM). Figure corresponds to Figure 5a in the manuscript but for normal filtered instead of low-frequency-extension filtered data.

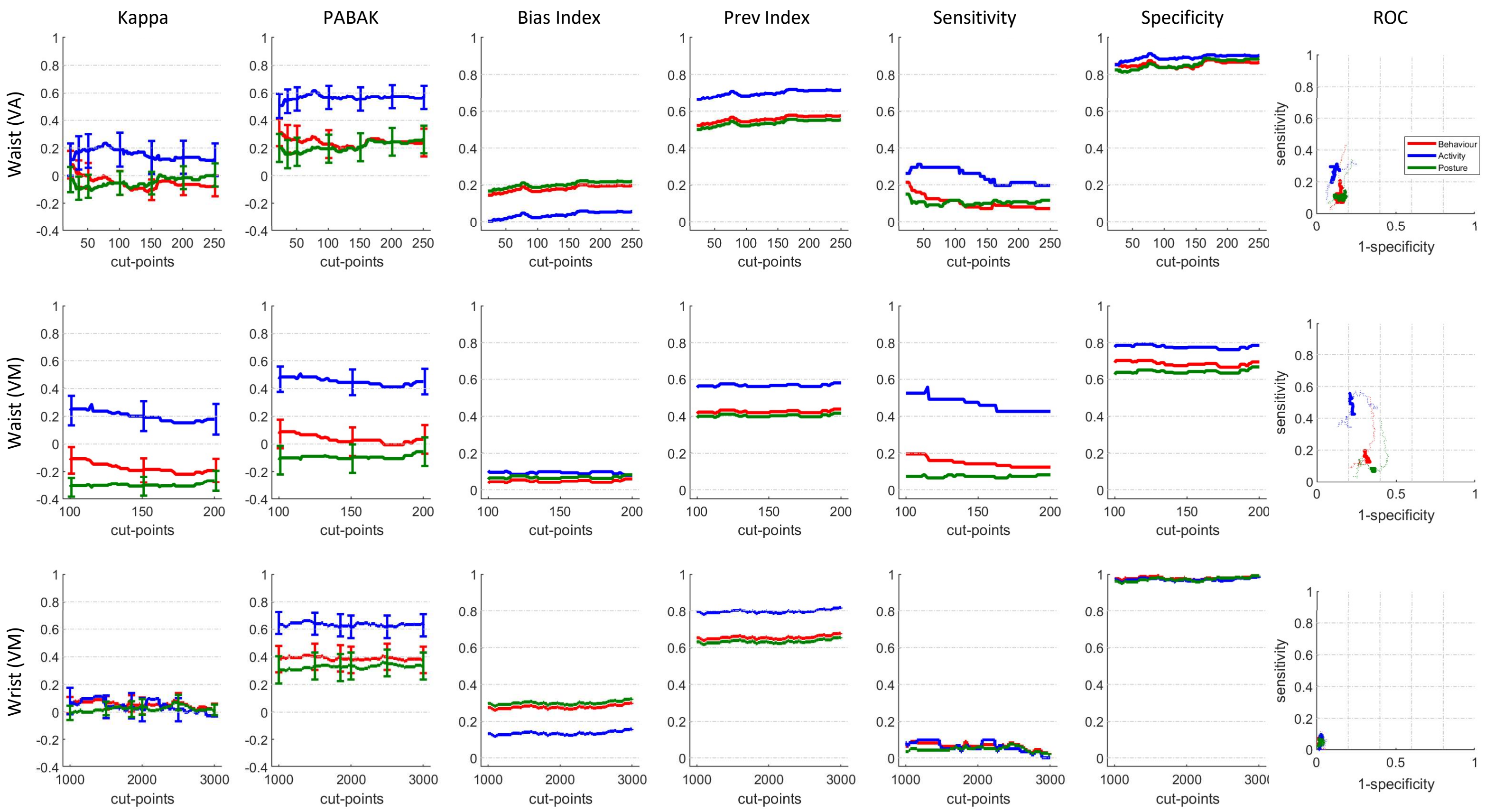

Additional File 1 - Figure 6: Kappa, Prevalence-adjusted-bias-adjusted-kappa (PABAK), bias and prevalence index, as well as sensitivity, specificity, and ROC curve to discriminate tasks effects on activity. Normal filtered data shown for the waist vertical axis (VA), vector magnitude (VM) and wrist VM. The Kappa and PABAK error bars denote the 95% confidence interval of commonly used counts-per-minute (cpm) cut-points (cut-point indicated on x-axis, for waist VA and wrist VM additionally for 22 and 35 cpm and 1'853 cpm, respectively). The dotted lines show the ROC for lower (down to 0) and higher cut-points (up to 500 and 750 for waist VA and VM and 15'000 for wrist VM). Figure corresponds to Figure 5b in the manuscript but for normal filtered instead of low-frequency-extension filtered data.

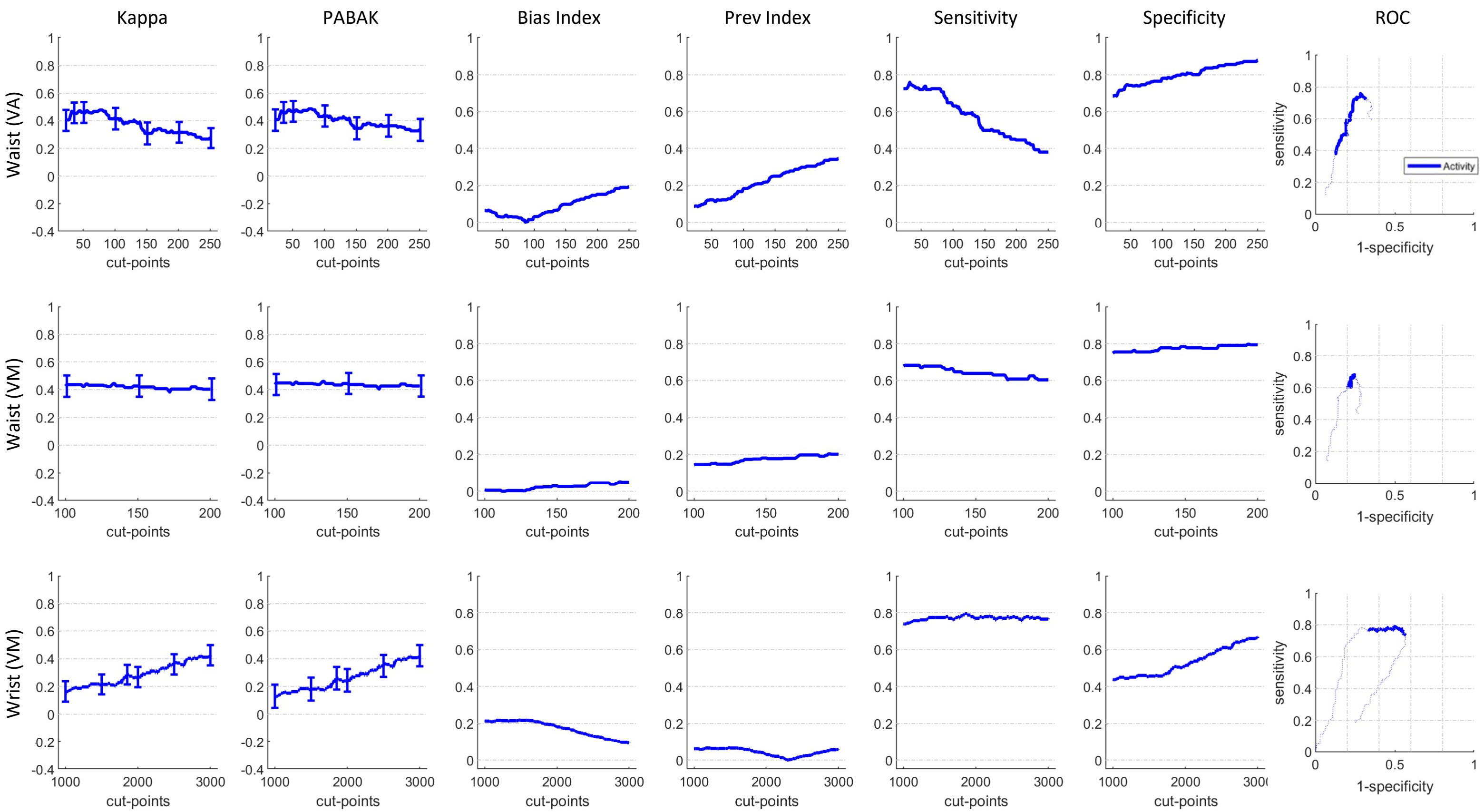

Supplement: Supplementary file 1 — Additional file 1. Normal Filtered Data, showing the data presented in Table 3 and Figs. 2, 3, 4 and 5 for the normal filtered instead of the low-frequency-extension filtered data, including the prevalence-adjusted-bias-adjusted-kappa and the sensitivity and specificity. [file 12889_2021_10387_MOESM1_ESM.pdf]
