# Supplementary material for: Concurrent and discriminant validity of ActiGraph waist and wrist cut-points to measure sedentary behaviour, activity level, and posture in office work
Source: BMC Public Health. 2021 Feb 12;21:345. doi: 10.1186/s12889-021-10387-7 (PMC7881682; doi:10.1186/s12889-021-10387-7)

Additional File 2 - Figure 1: Kappa and ROC-curve to detect sedentary behaviour (SB), minimal-intensity physical activity (minPA), and sitting for the all minutes approach and the individual minutes (grey). Data shown for the waist vertical axis (VA), vector magnitude (VM) and wrist VM. The kappa error bars denote the 95% confidence interval of commonly used counts-per-minute (cpm) cut-points (cut-point indicated on x-axis, for waist VA and wrist VM additionally for 22 and 35 cpm and 1'853 cpm, respectively). The dotted lines show the ROC for lower (down to 0) and higher cut-points (up to 500 and 750 for waist VA and VM and 15'000 for wrist VM). Figure corresponds to Figure 2 in the manuscript but includes the results for the individual minutes.

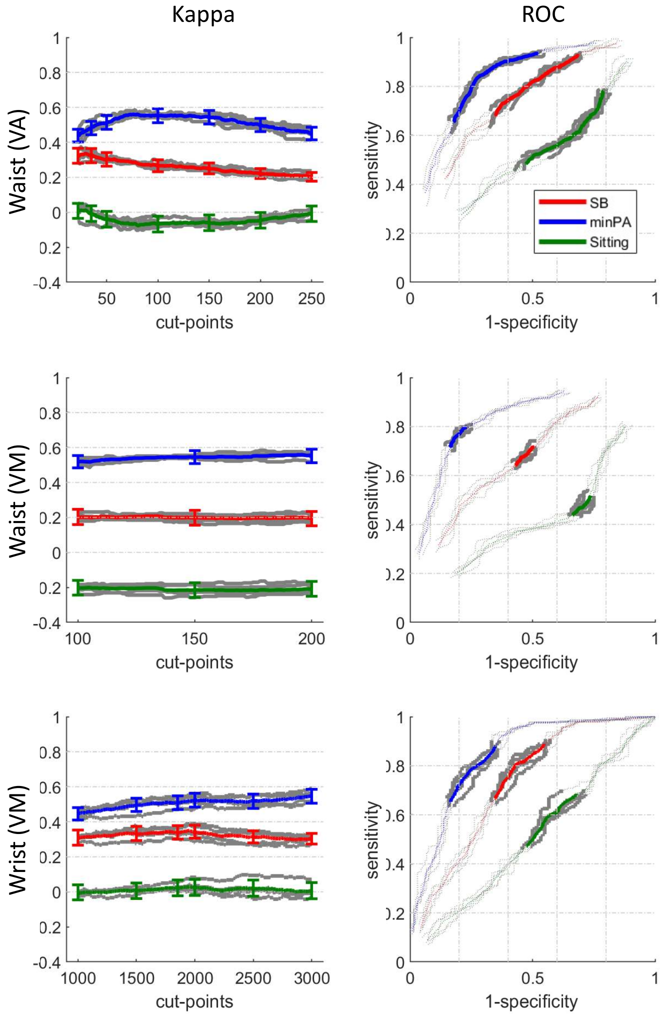

Additional File 2 - Figure 2: Kappa and ROC-curve to discriminate sedentary behaviour (SB), minimal-intensity physical activity (minPA), and sitting for the all minutes approach and the individual minutes (grey). Data shown for the waist vertical axis (VA), vector magnitude (VM) and wrist VM. The kappa error bars denote the 95% confidence interval of commonly used counts-per-minute (cpm) cut-points (cut-point indicated on x-axis, for waist VA and wrist VM additionally for 22 and 35 cpm and 1'853 cpm, respectively). The dotted lines show the ROC for lower (down to 0) and higher cut-points (up to 500 and 750 for waist VA and VM and 15'000 for wrist VM). Figure corresponds to Figure 4 in the manuscript but includes the results for the individual minutes.

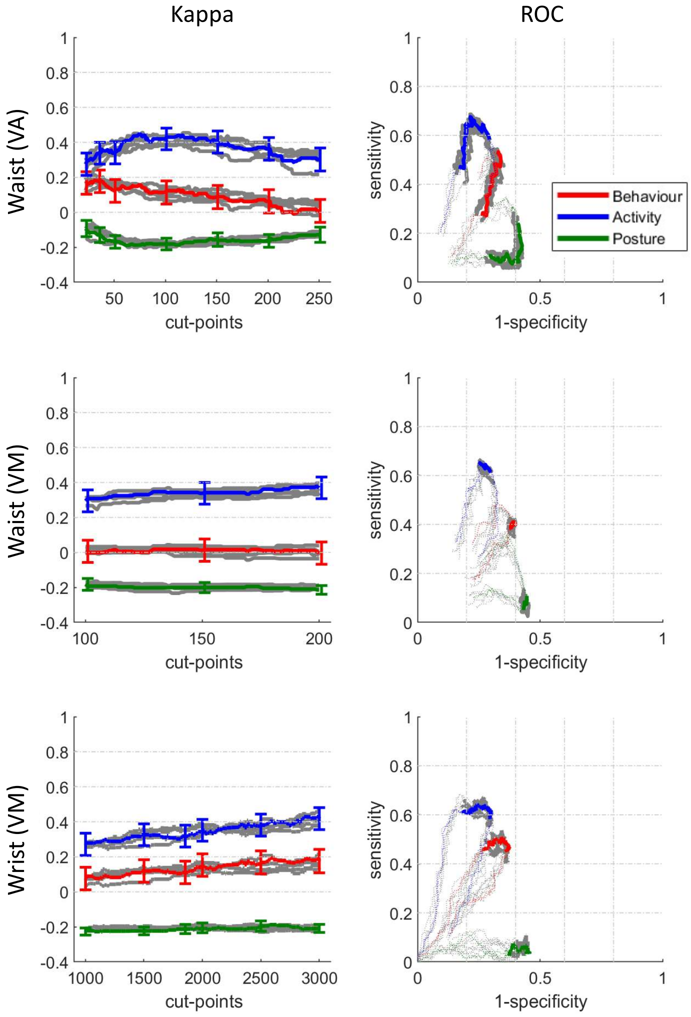

Supplement: Supplementary file 2 — Additional file 2. All minutes versus single minutes, comparing the data of the all minutes approach as presented in Figs. 2 and 4 with the analysis of each single minute. [file 12889_2021_10387_MOESM2_ESM.pdf]
