# Supplementary material for: Concurrent and discriminant validity of ActiGraph waist and wrist cut-points to measure sedentary behaviour, activity level, and posture in office work
Source: BMC Public Health. 2021 Feb 12;21:345. doi: 10.1186/s12889-021-10387-7 (PMC7881682; doi:10.1186/s12889-021-10387-7)

Additional File 3 - Figure 1: Prevalence-adjusted-bias-adjusted-kappa (PABAK), bias and prevalence index, as well as sensitivity and specificity to detect sedentary behaviour (SB), minimal-intensity physical activity (minPA), and sitting. Data shown for the waist vertical axis (VA), vector magnitude (VM) and wrist VM. The PABAK error bars denote the 95% confidence interval of commonly used counts-per-minute (cpm) cut-points (cut-point indicated on x-axis, for waist VA and wrist VM additionally for 22 and 35 cpm and 1'853 cpm, respectively). Same data as in Figure 2 in the manuscript shown.

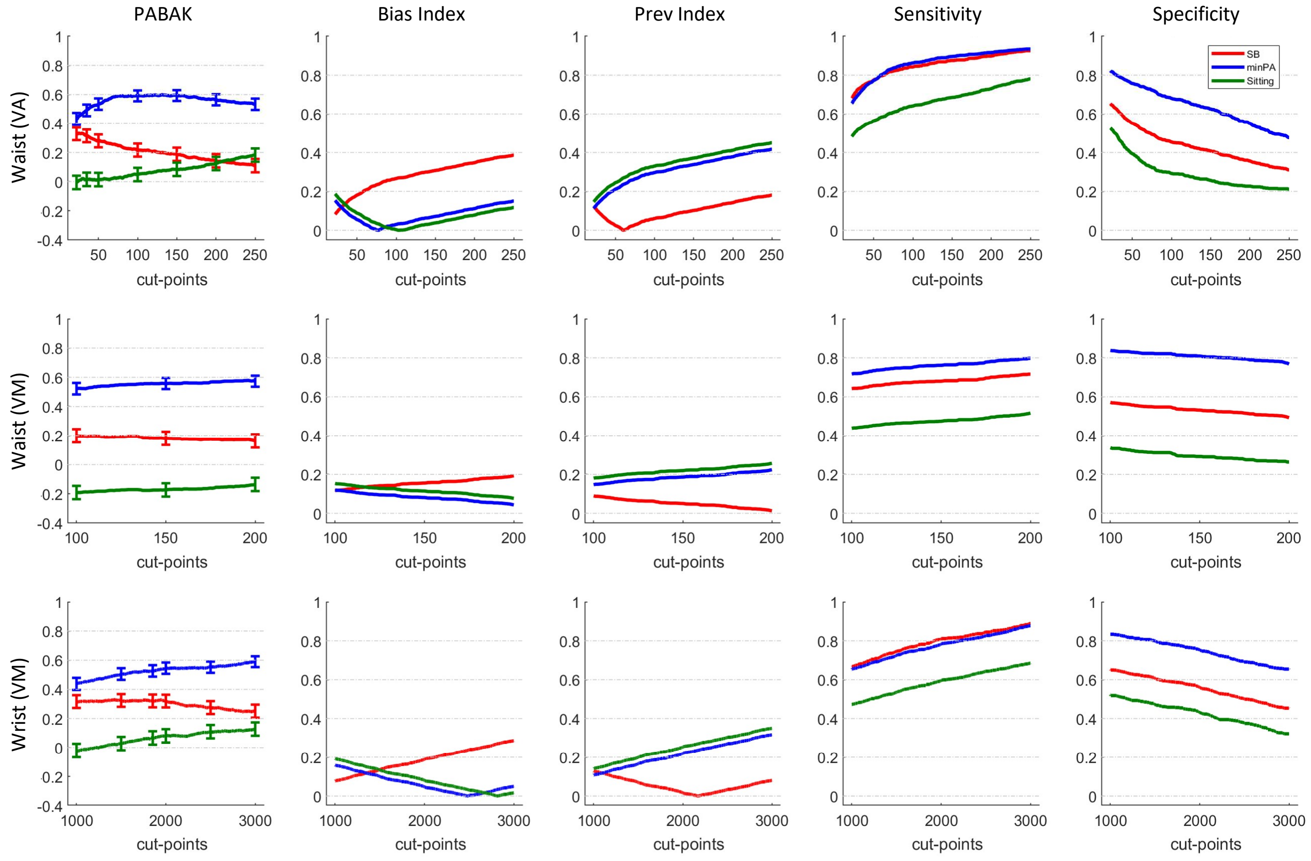

Additional File 3 - Figure 2: Prevalence-adjusted-bias-adjusted-kappa (PABAK), bias and prevalence index, as well as sensitivity and specificity to detect minimal-intensity physical activity for each workplace. Data shown for the waist vertical axis (VA), vector magnitude (VM) and wrist VM. The PABAK error bars denote the 95% confidence interval of commonly used counts-per-minute (cpm) cut-points (cut-point indicated on x-axis, for waist VA and wrist VM additionally for 22 and 35 cpm and 1’853 cpm, respectively). Same data as in Figure 3a in the manuscript shown.

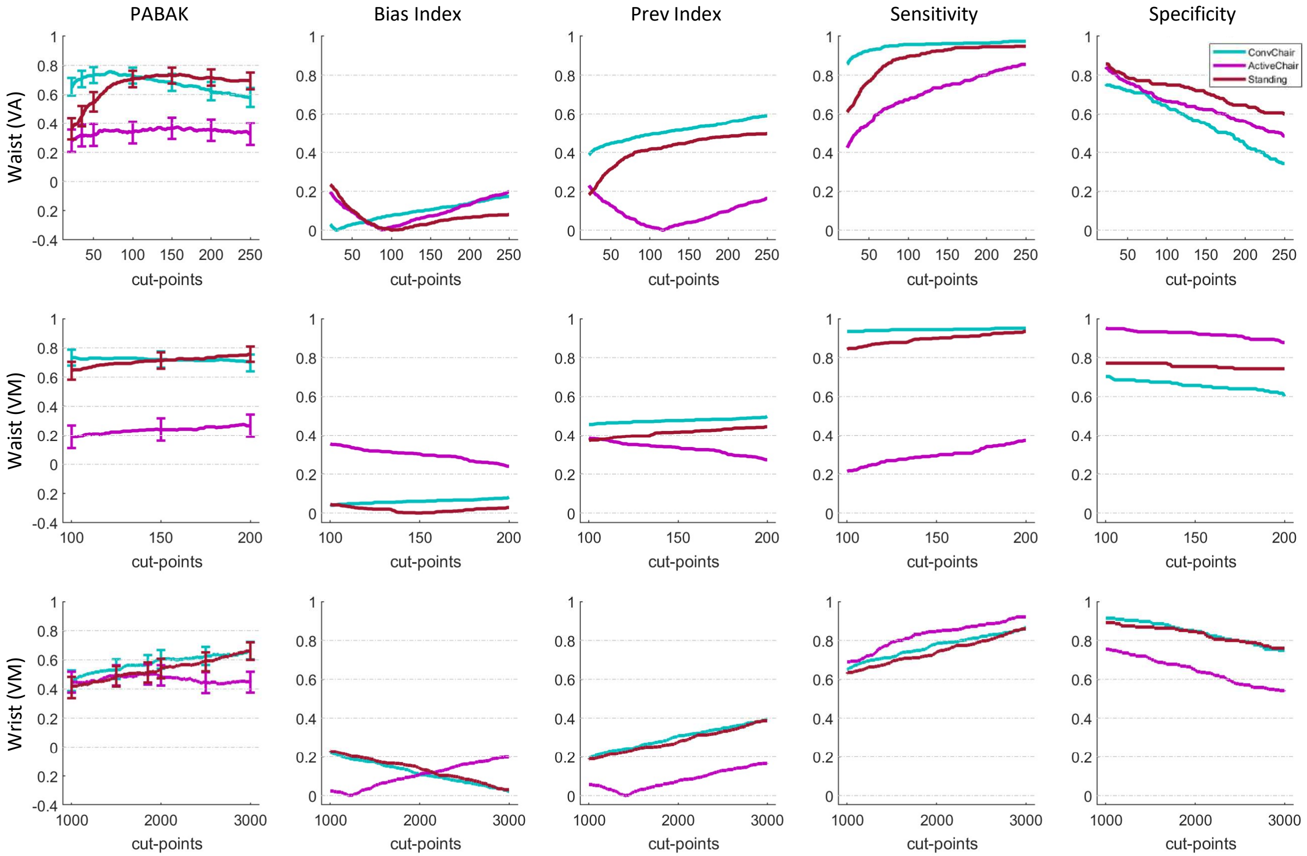

Additional File 3 - Figure 3: Prevalence-adjusted-bias-adjusted-kappa (PABAK), bias and prevalence index, as well as sensitivity and specificity to detect minimal-intensity physical activity for each task. Data shown for the waist vertical axis (VA), vector magnitude (VM) and wrist VM. The PABAK error bars denote the 95% confidence interval of commonly used counts-per-minute (cpm) cut-points (cut-point indicated on x-axis, for waist VA and wrist VM additionally for 22 and 35 cpm and 1'853 cpm, respectively). Same data as in Figure 3b in the manuscript shown.

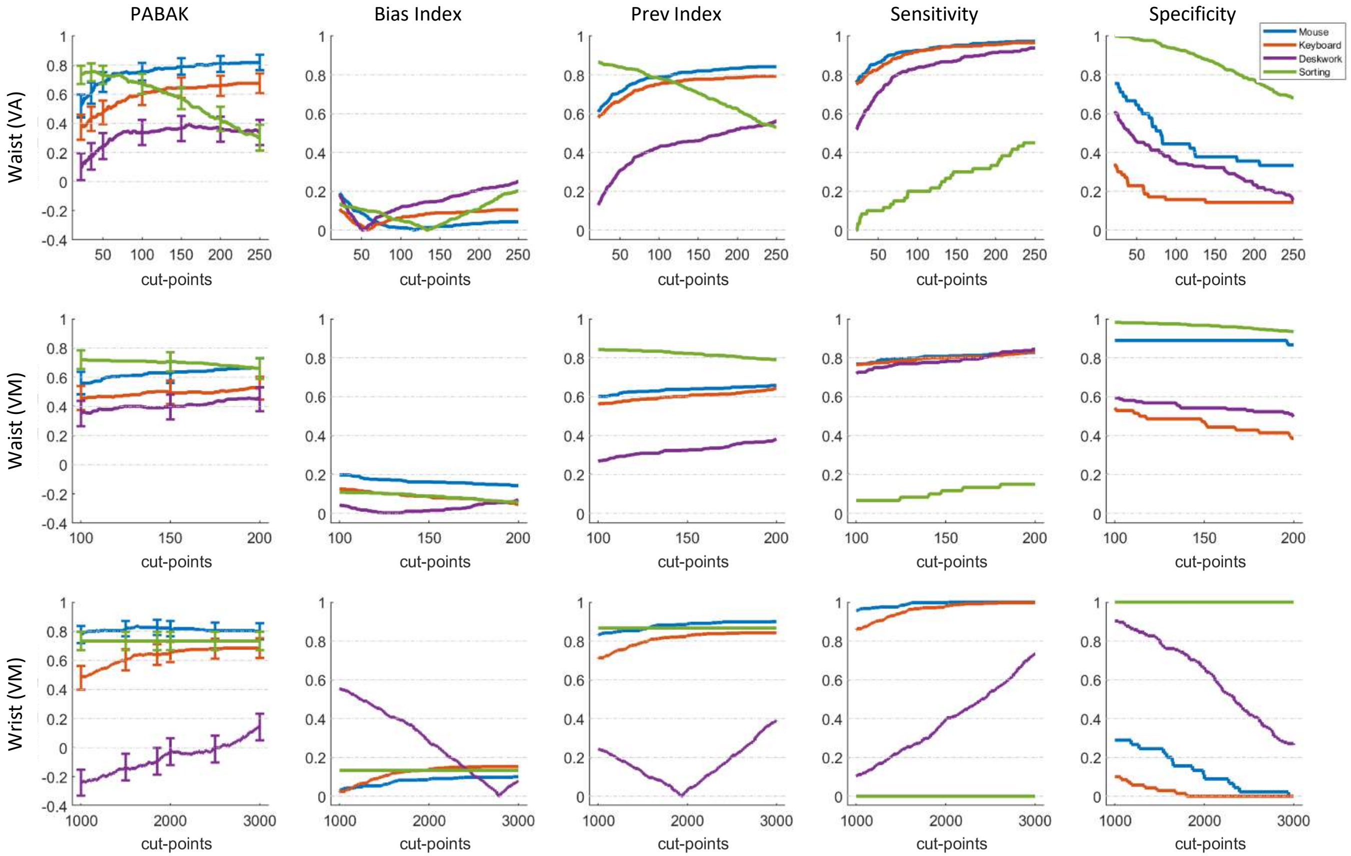

Additional File 3 - Figure 4: Prevalence-adjusted-bias-adjusted-kappa (PABAK), bias and prevalence index, as well as sensitivity and specificity to discriminate sedentary behaviour (SB), activity, and posture. Data shown for the waist vertical axis (VA), vector magnitude (VM) and wrist VM. The PABAK error bars denote the 95% confidence interval of commonly used counts-per-minute (cpm) cut-points (cut-point indicated on x-axis, for waist VA and wrist VM additionally for 22 and 35 cpm and 1'853 cpm, respectively). Same data as in Figure 4 in the manuscript shown.

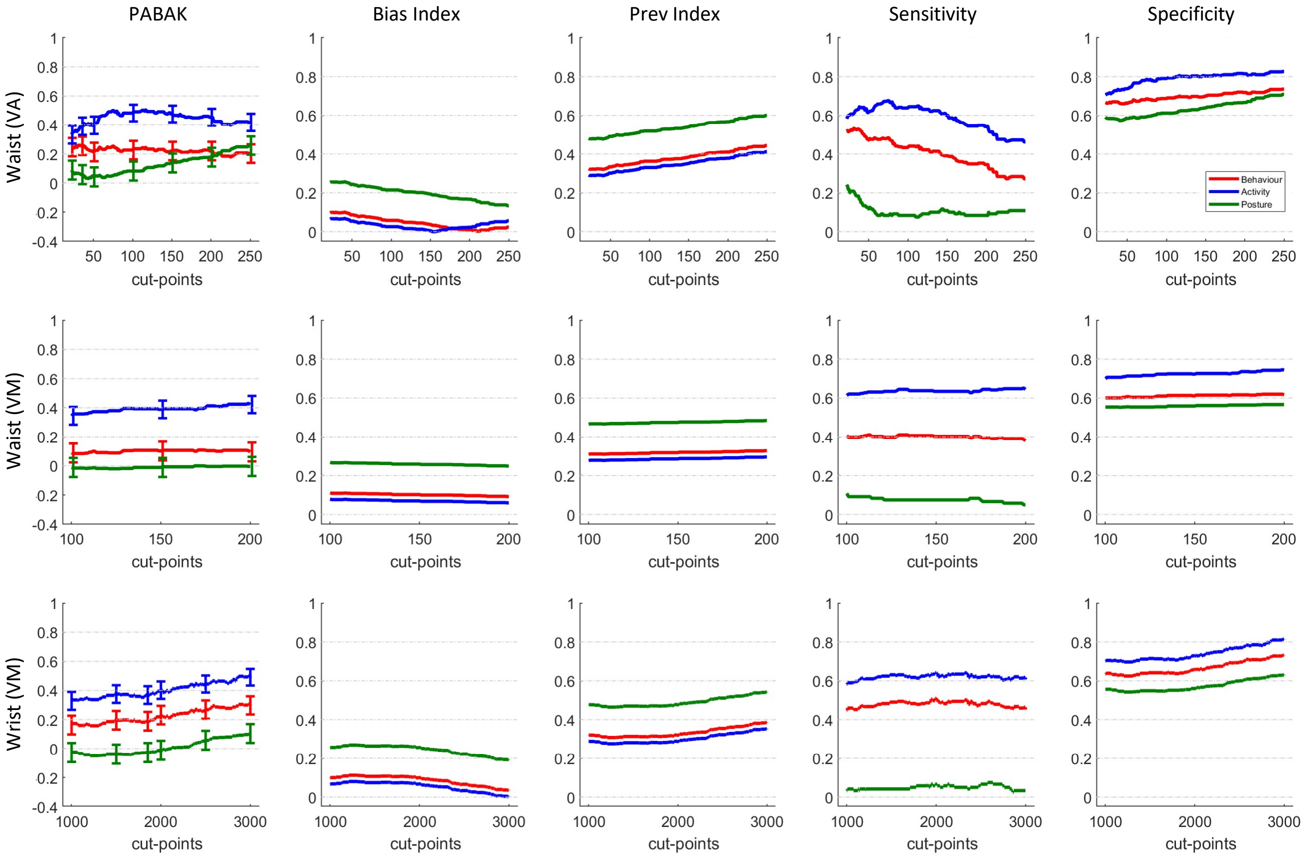

Additional File 3 - Figure 5: Prevalence-adjusted-bias-adjusted-kappa (PABAK), bias and prevalence index, as well as sensitivity and specificity to discriminate workplace effects on sedentary behaviour (SB), activity, and posture. Data shown for the waist vertical axis (VA), vector magnitude (VM) and wrist VM. The PABAK error bars denote the 95% confidence interval of commonly used counts-per-minute (cpm) cut-points (cut-point indicated on x-axis, for waist VA and wrist VM additionally for 22 and 35 cpm and 1’853 cpm, respectively). Same data as in Figure 5a in the manuscript shown.

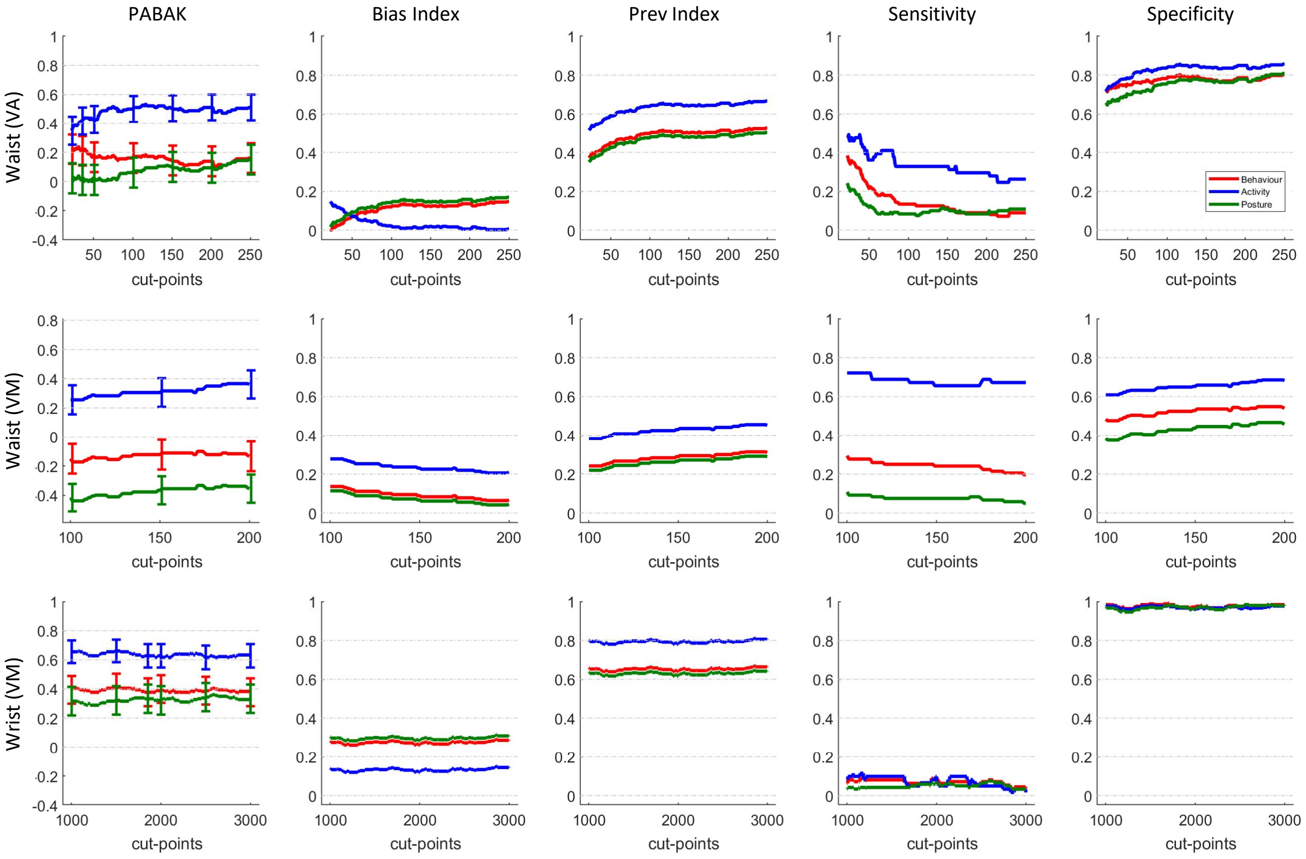

Additional File 3 - Figure 6: Prevalence-adjusted-bias-adjusted-kappa (PABAK), bias and prevalence index, as well as sensitivity and specificity to discriminate task effects on activity. Data shown for the waist vertical axis (VA), vector magnitude (VM) and wrist VM. The PABAK error bars denote the 95% confidence interval of commonly used counts-per-minute (cpm) cut-points (cut-point indicated on x-axis, for waist VA and wrist VM additionally for 22 and 35 cpm and 1’853 cpm, respectively). Same data as in Figure 5b in the manuscript shown.

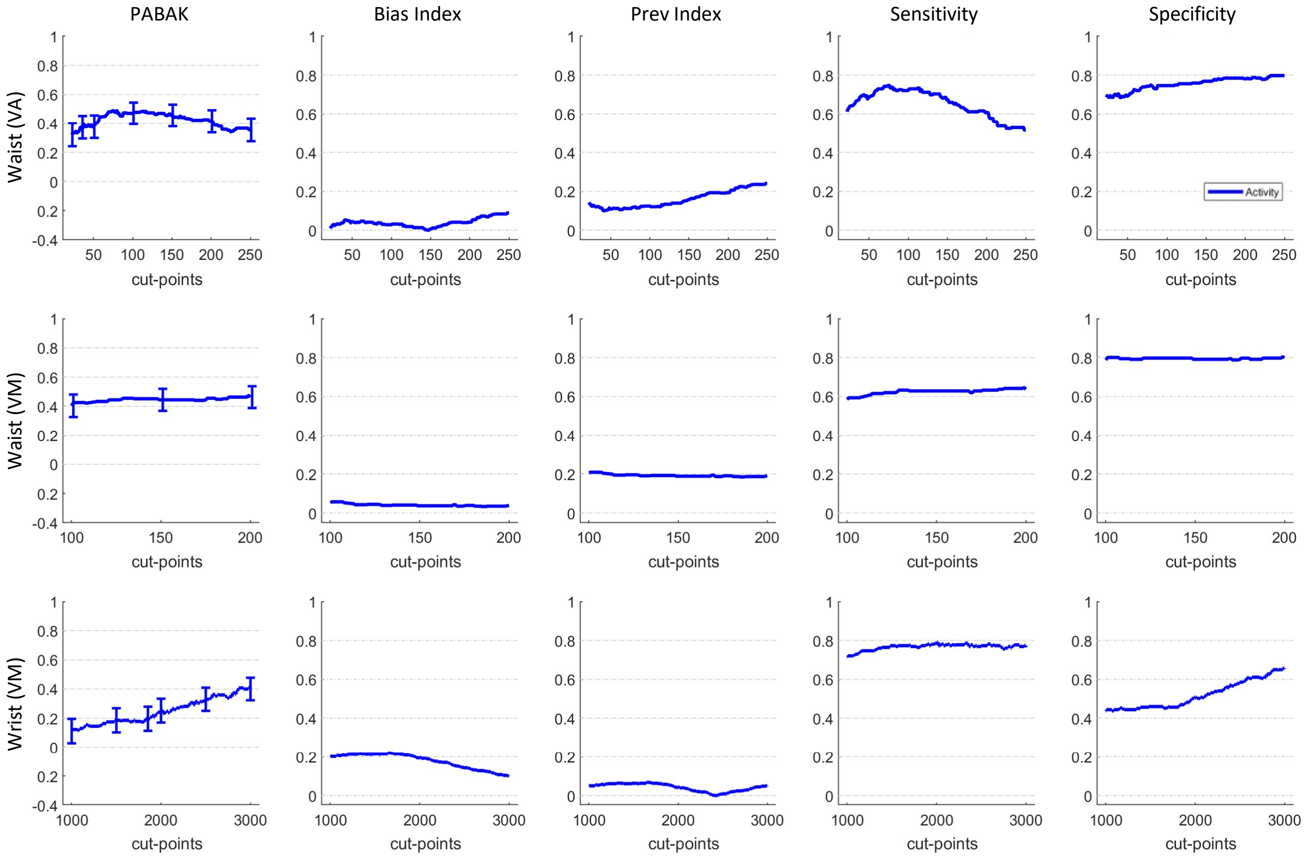

Supplement: Supplementary file 3 — Additional file 3. Prevalence-adjusted-bias-adjusted-kappa and sensitivity and specificity, shown for each figure in the manuscript. [file 12889_2021_10387_MOESM3_ESM.pdf]
